# Traditional uses, pharmacological activities, and phytochemical constituents of the genus *Syzygium*: A review

**DOI:** 10.1002/fsn3.2797

**Published:** 2022-03-04

**Authors:** A. B. M. Neshar Uddin, Farhad Hossain, A. S. M. Ali Reza, Mst. Samima Nasrin, A. H. M. Khurshid Alam

**Affiliations:** ^1^ 54495 Department of Pharmaceutical Sciences North South University Dhaka Bangladesh; ^2^ 118862 Department of Pharmacy Faculty of Science and Engineering International Islamic University Chittagong Chittagong Bangladesh; ^3^ 118869 Department of Pharmacy University of Rajshahi Rajshahi Bangladesh

**Keywords:** Myrtaceae, pharmacological activities, phytochemical constituents, *Syzygium*, traditional use

## Abstract

The genus *Syzygium* comprises 1200–1800 species that belong to the family of Myrtaceae. Moreover, plants that are belonged to this genus are being used in the traditional system of medicine in Asian countries, especially in China, India, and Bangladesh. The aim of this review is to describe the scientific works and to provide organized information on the available traditional uses, phytochemical constituents, and pharmacological activities of mostly available species of the genus *Syzygium* in Bangladesh. The information related to genus *Syzygium* was analytically composed from the scientific databases, including PubMed, Google Scholar, Science Direct, Web of Science, Wiley Online Library, Springer, Research Gate link, published books, and conference proceedings. Bioactive compounds such as flavanone derivatives, ellagic acid derivatives and other polyphenolics, and terpenoids are reported from several species of the genus *Syzygium*. However, many members of the species of the genus *Syzygium* need further comprehensive studies regarding phytochemical constituents and mechanism‐based pharmacological activities.

## Graphical abstract

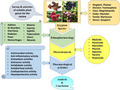

## 1 INTRODUCTION

Plant is an essential source of medicine and plays a vital role in world health for its therapeutic or curative aids which have attained a commanding role in health system all over the world (Akkol, Tatlı, et al., 2021; Fernández et al., 2021; Hossain et al., 2020; Rahman et al., 2021). This comprises medicinal plants not only important for the treatment of diseases but also as potential material for maintaining good health and conditions. Better cultural acceptability, better compatibility and adaptability with the human body, and lesser side effects of plants made many countries in the world to depend on herbal medicine for their primary health care (Bari et al., 2021; CHOWDHURY et al., 2021; Hoque et al., 2021). For centuries, plants are being widely used for their natural resources isolated from various parts of a plant and have been used in the treatment of human diseases. Moreover, some of these plants produce edible fruits, while others simply produce dazzling flowers, which are so attractive and important economically as well. The knowledge of traditional medicine supports all other systems of medicine such as Ayurveda, Siddha, Unani, and even modern medicine. Plant‐based systems always play an essential role in health care, and their use by different cultures has been extensively documented. The number of plants used for treatment is estimated to be 20,000 named as medicinal plants. The studies on medicinal plants and active substances derived from these have increased the interest in plants for modern medicine in recent years (F. Hossain et al., 2021; S. Hossain et al., 2021; Sinan et al., 2021; Tallei et al., 2021).

The genus *Syzygium* is one of the genera of the myrtle family Myrtaceae comprising 1200–1800 species spread out over the world (B. Ahmad et al., 2016; Reza et al., 2021). The genus *Syzygium* (Myrtaceae) is named after a Greek word meaning “coupled,” an illusion to the paired branches and leaves (Nigam & Nigam, 2012). It has an extensive range that spread out from Africa and Madagascar, Asia throughout the Pacific (Tuiwawa et al., 2013), and highest levels of diversity ensue from Malaysia to Australia, where numerous species are very poorly known and countless species have not been portrayed taxonomically. These species are abundant components in the upper and medium strata of rainforests of the eastern Australia (Hyland, 1983). It is the biggest woody genus of the flowering plants in the world (B. Ahmad et al., 2016). Majority of the species of *Syzygium* genus vary from medium to large evergreen trees. Some of the species produce edible fruits (e.g., *S*. *jambos, S. fibrosum*), which are eaten freshly or commercially used to form them in jam and jellies (Table 1). The genus *Syzygium* has also a culinary use such as clove of some species, for example, *S*. *aromaticum* (B. Ahmad et al., 2016), whose unopened flower buds are used as a spice which is most important economically. Few species are used as flavoring agents for their attractive glossy foliage, while other species look ornamented. Many species of the genus *Syzygium* are known for their traditional use in various diseases. *S*. *aromaticum's* essential oil (CEO) is traditionally used in the treatment of burns and wounds, and as a pain reliever in dental care as well as treating tooth infections and toothache (Batiha et al., 2020). *S. cordatum* and *S. guineese* are used in abdominal pain, indigestion, and diarrhea (N. Dharani, 2016). *S. cumini* is used in diarrhea, dysentery, menorrhagia, asthma, and ulcers (Jadhav et al., 2009). *S*. *jambos* (L.) is traditionally used to treat hemorrhages, syphilis, leprosy, wounds, ulcers, and lung diseases (Reis et al., 2021). *S*. *malaccense* (L.) is used to treat mouth ulcers and irregular menstruation; *S*. *samarangense* flowers are used to treat diarrhea and fever; and *S*. *suboriculare* is used to treat coughs and colds, diarrhea, and dysentery (IE Cock & Cheesman, 2018). *S*. *caryophyllatum*, *S*. *cumini*, *S*. *malaccense*, and *S*. *samarangens* are used to treat diabetes mellitus (Ediriweera & Ratnasooriya, 2009).

**TABLE 1:** A list of selected plants belonging to the *Syzygium* genus, including the plant parts with their traditional uses

Plant Name	Part	Indications	References
*Syzygium alternifolium* (WT.)* *WALP	Leaf (juice), tender shoots (pulp)	Bacillary dysentery	(Yugandhar et al., 2015)
	Fruits (powder)	Diarrhea and diabetes	(Yugandhar et al., 2015)
*Syzygium anisatum* (Vicfkery) Craven & Biffen	Leaf (EO)	Antiseptic	(Bryant & Cock, 2016)
*Syzygium aqueum* (Burm.f.) Alston	Leaf	Antibiotic and childbirth pain	(T. Manaharan et al., 2013)
*Syzygium aromaticum* (L) Merr. & Perry.	Flower bud	Aromatic stomachic, anti‐inflammatory agent, deodorant, disinfectant	(Kasai et al., 2016)
	Flower bud	Toothache, gum inflammation, coughs, colds, neuralgic pain, and rheumatism	(N. Dharani, 2016)
*Syzygium australe* (H.L. Wendl. Ex Link) B. Hyland	_	Fungal skin infections	(Noé et al., 2019)
*Syzygium campanulatum* Korth	_	Stomach pain	(Abdul Hakeem Memon et al., 2020)
*Syzygium calophyllifolium* Walp.	Leaf	Skin diseases	(Chandran et al., 2016)
	Fruit and bark	Aching tooth and inflammation	(Chandran et al., 2016)
*Syzygium caryophyllatum* (L.) Alston	_	Diabetes mellitus	(Ediriweera & Ratnasooriya, 2009)
*Syzygium cordatum* Hochst ex C Krauss	Leaf, root, and bark	Stomachaches, abdominal pains, indigestion, diarrhea, diabetes, and venereal diseases	(N. Dharani, 2016)
	Leaf, root, bark and fruit	Gastrointestinal disorders, burns, sores, wounds, colds, cough, respiratory complaints, sexually transmitted infections (STIs), tuberculosis, fever, and malaria	(Maroyi, 2018)
*Syzygium cumini* (L.) Skeels	Fruit	Cough, diabetes, dysentery, inflammation, ringworm, and gastrointestinal complaints	(N. Dharani, 2016)
	Leaf	Diabetes, diarrhea, leucorrhea, and stomach pains	(N. Dharani, 2016)
	Stem bark	Bleeding gums, venereal ulcers, dysentery, and fresh wounds	(N. Dharani, 2016)
*Syzygium densiflorum* Wall. ex Wt. & Arn.	Leaf and ripened fruit	Diabetes	(Krishnasamy et al., 2016)
*Syzygium fruticosum* (Roxb.) DC.	Leaf (juice)	Blood dysentery	(A. H. M. M. Rahman & Khanom, 2013)
	‐	Stomachic, diabetes and bronchitis	(Chadni et al., 2014)
*Syzygium formosum* (Wall.) Masam	Leaf	Allergy or skin rash	(Duyen Vu et al., 2019)
*Syzygium grande* (Wight) Walp.	_	Diabetic‐related complications	(Huong et al., 2017)
*Syzygium gratum* (Wight) S.N. Mitra	_	Dyspepsia, indigestion, peptic ulcer, diarrhea, bacterial infection, asthma, and cardiovascular diseases	(Senggunprai et al., 2010)
*Syzygium guineense* (Willd.) DC.	Root and stem bark	Stomachaches and infertility	(N. J. P. J. K. Dharani, 2016)
	Leaf (decoction)	Intestinal parasites, stomachache, diarrhea and ophthalmia	(N. J. P. J. K. Dharani, 2016)
*Syzygium jambos* L. (Alston)	_	Hemorrhages, syphilis, leprosy, wounds, ulcers, and lung diseases	(Reis et al., 2021)
*Syzygium lineatum* (DC.) Merr. & L.M. Perry	_	Cancer	(Castillo et al., 2018)
*Syzygium luehmannii* (F. Muell.) L.A.S. Johnson	_	Fungal skin infections	(Noé et al., 2019)
*Syzygium malaccense* (L.)Merr. & L.M. Perry	Bark (decoction)	Mouth ulcers	(IE Cock & Cheesman, 2018)
	Leaf	Irregular menstruation	(IE Cock & Cheesman, 2018)
*Syzygium myrtifolium* Walp.	_	Stomach aches;	(Kusriani et al., 2019)
*Syzygium mundagam* (Bourd.) Chitra	_	Diabetes	(Chandran et al., 2017)
*Syzygium nervosum* A. Cunn.ex DC.	Leaf and flower bud	Abdominal pain, diarrhea, wounds, itchy sores and acne	(Pham et al., 2020)
	Leaf and bark	Skin ulcers, scabies, and other skin diseases	(Pham et al., 2020)
	Leaf	Diarrhea, pimples, and breast inflammation	(Pham et al., 2020)
*Syzygium paniculatum* Gaertn.	_	Diabetes	(Konda et al., 2019)
*Syzygium polyanthum* (Wight) Walp.	Leaf	diabetes mellitus, hypertension, gastritis, ulcers, diarrhea, skin diseases diabetes mellitus, hypertension, gastritis, ulcers, diarrhea, skin diseases Diabetes mellitus, hypertension, gastritis, ulcers, diarrhea, and skin diseases	(Ismail & Ahmad, 2019)
*Syzygium samarangense* (Blume) Merr. and L.M. Perry	Flower	Diarrhea and fever	(IE Cock & Cheesman, 2018)
*Syzygium suboriculare* (Benth.) T.G. Hartley & L.M. Perry	_	Cough, cold, diarrhea, and dysentery	(IE Cock & Cheesman, 2018)
*Syzygium zeylanicum* (L.) DC.	Leaf (extract)	Joint pain, headache, arthritis, and fever	(Anoop et al., 2015)
	Stem bark	Diabetes mellitus	(Shilpa & Krishnakumar, 2015)

Although many species of *Syzygium* genus are used as traditional medicine (Table 1), in this review, we tried to provide an overview on the phytochemical constituents and pharmacological activities of *Syzygium* species for the development of evidence‐based medicines. It is important to analyze the critique of these species in relation to current knowledge of bioactive compounds and biological activities, which may reduce the gaps between the traditional knowledge and evidence‐based research in future.

## 2 BOTANICAL DESCRIPTION

### 2.1 Nomenclature

*Syzygium* is an entirely old world genus. In past, many *Syzygium* species were originally described in *Eugenia L*. or *Jambosa Adans*. Taxonomic confusion in *Eugenia* and *Syzygium* resulted from the considerable overlap of macro‐ and micromorphological characters. Currently, it is clear that these genera are significantly different. Recent molecular evidence supports a scenario in which these two genera are in fact independent lineages (Widodo, 2011).

### 2.2 Phylogeny

Based upon evolutionary relationships as inferred from investigation of nuclear and plastid DNA sequence data, which is reinforced by morphological evidence presented an infrageneric classification of *Syzygium*. Six subgenera and seven sections were recognized. The six subgenera are *Syzygium, Acmena, Sequestratum, Perikion, Anetholea, Wesa,* and the seven sections are *Gustavioides, Monimioides, Glenum, Waterhousea, Agaricoides, Acmena, Piliocalyx* (Craven, Biffin, & Plants, 2010).

### 2.3 Morphology

*Syzygium* is mostly found in tropical or subtropical vegetation, ranging from lowland to montane rainforest, swamp, ultramafic forest, savannah to limestone forest, and also most common tree genera in the forest ecosystem. Some species arise in specified habitat such as along river or on ultramafic or limestone soil. *Syzygium* are morphologically categorized by a narrow leaf, short petiole, and flexible twig, and leaves are crowded at twig ends. *Syzygium* commonly blooms in masses in tropical rainforest. It is also important as a food resource for birds, insects, and mammals (Soh et al., 2017).

### 2.4 Geographical distribution

The genus is native to Bangladesh, India, Pakistan, Sri Lanka, Malaysia, the Philippines, Myanmar, China, and Thailand. It is considered as exotic in Australia, Algeria, Bahamas, Colombia, Ghana, Guatemala, Grenada, Guyana, Jamaica, Kenya, Mexico, Nepal, the Netherlands, Panama, South Africa, and United States of America (Nigam & Nigam, 2012).

## 3 METHODOLOGY

The present review article reports every aspect of the plant including its traditional uses, phytochemical constituents, and pharmacological activities from the genus *Syzygium* considering the literatures published prior to September 2020. All the available information on the genus *Syzygium* was conducted through searching variant scientific electronic databases, including PubMed, Google Scholar, Science Direct, Web of Science, Wiley Online Library, Springer, and Research Gate link, and additional information was conducted from other sources such as book and journals written in English. The literature searched is characterized under detail headings in individual section from the databases.

## 4 PHYTOCHEMICAL CONSTITUENTS

### 4.1 Flavonoids

**FIGURE 1:**
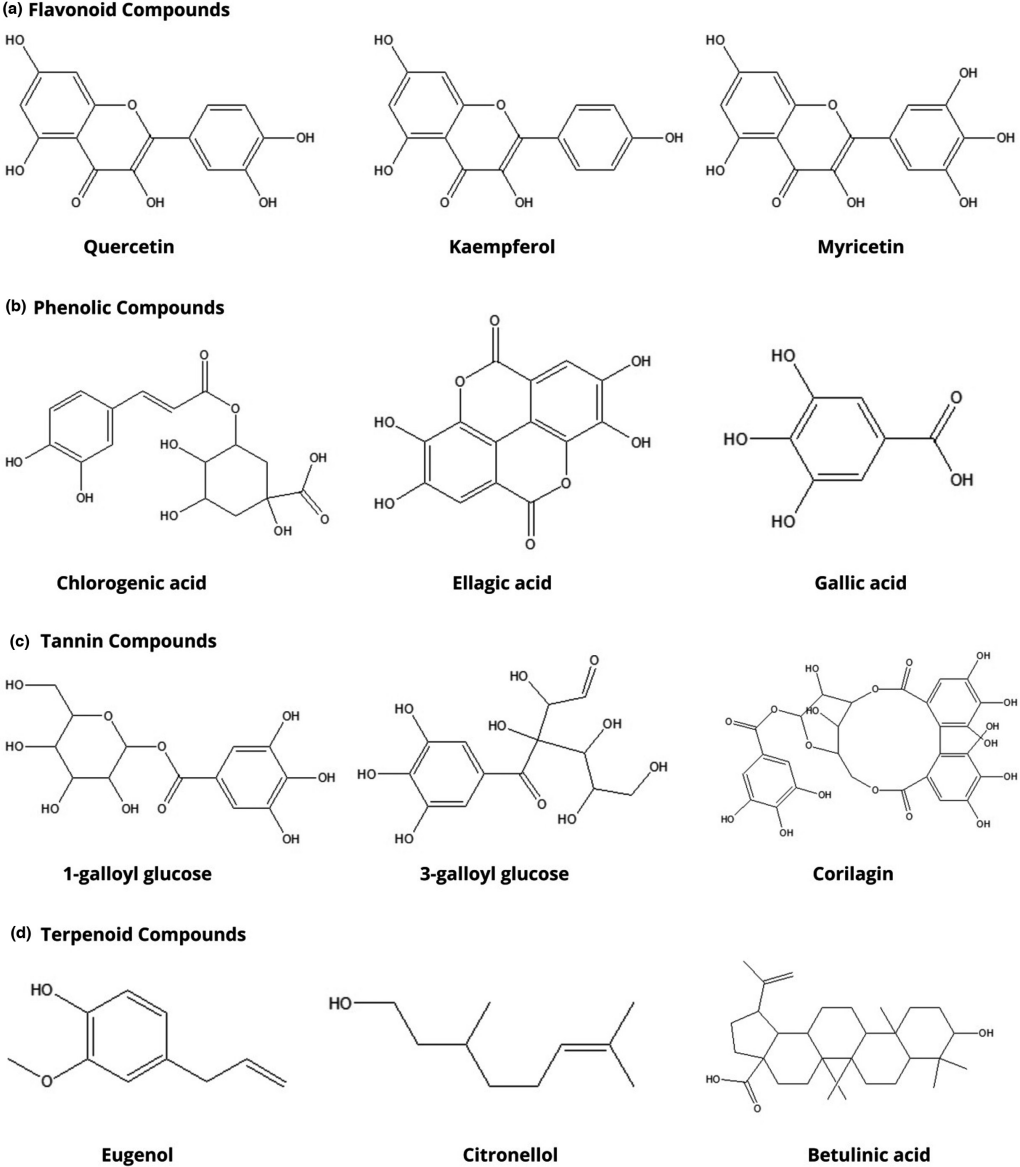
Different types of compounds isolated from *Syzygium* genus

Flavonoids are vital group of naturally occurring polyphenolic compounds having an antioxidant, anti‐inflammatory, antidiabetic, antiallergic activities, while some other flavonoid compounds exhibit potential antiviral activity (S. Ahmed et al., 2020; M. S. Islam et al., 2021; Karak & research, 2019). Methanol extract of *S. aqueum* leaves contained a number of 87 different compounds rich in flavonoids, for example, myricetin rhamnoside, myrigalone‐G pentoside, quercetin galloyl‐pentoside, cryptostrobin, in which myrigalone‐B and myrigalone‐G were the major flavonoid compounds (A. A. Ahmed et al., 2021; Sobeh, Mahmoud, et al., 2018, 2018). Six flavonoids (e.g., 4‐hydroxybenzaldehyde, myricetin‐3‐O‐rhamnoside, europetin‐3‐O‐rhamnoside, phloretin, myrigalone‐G, and myrigalone‐B) were isolated from the ethanol leaf extracts of *S*. *aqueum*. Among them, myricetin‐3‐O‐rhamnoside and europetin‐3‐O‐rhamnoside showed antihyperglycemic activity (Küpeli Akkol et al., 2020; Thamilvaani Manaharan et al., 2012). *S. campanulatum* n‐hexane and methanol leaf extract contained two flavonoid compounds (2S)‐7‐hydroxy‐5‐methoxy‐6,8‐dimethyl flavanone and (S)‐5,7‐dihydroxy‐6,8‐dimethyl‐flavanone assessed in HPLC method, showing strong antiproliferative activity against human colorectal carcinoma (HCT 116) cells (Hossen et al., 2021; Memon et al., 2015). *S. corticosum* chloroform leaf extract contained 19 compounds. Among them, two compounds were flavonoids (e.g., sideroxylin, 2,3‐dihydrosideroxylin) obtained through chromatographic separation (Ren et al., 2018). *S. cumini* contained very few number of flavonoid compounds in various parts such as leaf extract (ellagic acid; caffeic acid), bark extract (quercetin; kaemferol; Figure 1), seed extract (quercetin; rutin), and flower extract (kaemferol, dihydromyricetin) of the plants (Chhikara et al., 2018). Gallic acid methyl ester, a compound identified and characterized from *S. fruticosum*, exhibited strong antibacterial activity, cytotoxic activity, higher ferrous reducing antioxidant and DPPH free radical scavenging activities (Nasrin et al., 2018). Ethanolic extract of *S. formosum* leaves contained 28 compounds. Among these, 11 compounds were flavonoids (e.g., catechin, myricetin, quercetin, kaempferol pentoside, etc.), determined by HPLC method (Duyen Vu et al., 2019). *S*. *samarangense* methanol leaf extract contained 92 compounds determined by LC‐ESI‐MS/MS method where major compounds were (epi)‐catechin‐(epi)‐gallocatechin, (epi)‐gallocatechin gallate, (epi)‐catechin‐afzelechin, myricetin pentoside, myricetin rhamnoside, guaijaverin, and isorhamnetin rhamnoside (Sobeh et al., 2018). Four flavonoids (e.g. 2′‐hydroxy‐4′,6′‐dimethoxy‐3′‐methylchalcone, 2′,4′‐dihydroxy‐6′‐methoxy‐3′,5′‐dimethylchalcone, 2′,4′‐dihydroxy‐6′‐methoxy‐3′‐methylchalcone, and 7‐hydroxy‐5‐methoxy‐6,8‐dimethylflavanone) isolated from the hexane extract of *S*. *samarangense* showed dose‐dependent (10–1000 µg/ml) spasmolytic activity, indicating the usefulness of the plant in the treatment of diarrhea (Ghayur et al., 2006; Table 2).

**TABLE 2:** A list of phytochemicals with their source of origin

Sl. No.	Plant Name	Extraction Solvent	Plant Parts	Chemical Compounds	References
01	*Syzygium alternifolium* (Wight) Walp.	Methanol	Stem Bark	Octamethylcyclotetrasiloxane, hexamethylcyclotrisiloxane; 1,5‐Diphenyl−2H−1,2,4‐triazoline−3‐thione; cyclopentasiloxane; and decamethyl	(Yugandhar & Savithramma, 2017)
		Methanol	Leaf	Diethoxydimethylsilane and acetaldehyde	(Yugandhar & Savithramma, 2017)
		Methanol	Fruits	Diethoxydimethylsilane; flavone, 2',5,6,6'‐tetramethoxy‐; 4H−1‐Benzopyran−4‐one	(Yugandhar & Savithramma, 2017)
02	*Syzygium anisatum* (Vicfkery) Craven & Biffen	Aqueous	Leaf	E‐anethole, methyl chavicol; Z‐ anethole; alpha‐Pinene; 1,8‐Cineole; alpha‐Farnesene; anisaldehyde	(Brophy & Boland, 1991)
03	*Syzygium aqueum* (Burm.f.) Alston	Methanol	Leaf	Myricetin rhamnoside; myrigalone‐G pentoside; quercetin galloyl‐pentoside; samarangenin A; (epi)‐gallocatechin gallate; digalloyl‐hexahydroxydiphenoyl (HHDP)‐hexoside; α‐selinene; β‐caryophyllene; and β‐selinene	(M. Sobeh et al., 2016; Sobeh, Mahmoud, et al., 2018, 2018)
04	*Syzygium aromaticum* (L) Merr. & Perry.	_	Leaf	Eugenol; β‐caryophyllene; 3‐hexen−1‐ol; and hexyl acetate;	(Kasai et al., 2016)
		_	Clove	Eugenol; eugenyl acetate; and β‐caryophyllene;	(Kasai et al., 2016)
		Distilled water	Seed	Eugenol acetate; β‐carophyllene; eugenin; eugenol; methyl salicylate	(Ajiboye et al., 2016)
05	*Syzygium arnottianum* Wall.ex Wight & Arn.	Methanol	Leaf	4‐Aminopyrimidine; Oxazole; Oct−3‐en−2‐yl ester Cyclopentanone	(Krishna & Mohan, 2012)
06	*Syzygium australe* (H.L. Wendl. Ex Link) B. Hyland	Methanol & aqueous	Leaf	1‐Vinylheptanol; 2‐ethyl−1‐hexanol; 2‐heptyl−1,3‐dioxolane; 1‐methyloctyl butyrate; linalool; and 1‐terpineol	(Noé et al., 2019)
07	*Syzygium benthamianum* (Duthie) Gamble	Ethyl acetate	Leaf	4‐(4‐Ethylcyclohexyl)−1‐pentyl‐Cyclohexene; Linoleic acid; 2,6,10,14,18‐Penta‐methyl−2,6,10,14,18‐eicosapentaene; 9,17‐Octadecadienal,(z)‐; Z,E−3,13‐Octadecadien−1‐ol; and 7‐Pentadecyne	(Kiruthiga et al., 2011)
		Double distilled water	Leaf	Sitosteryl acetate; stigmastan−3,5,22‐trien; 2,6‐dimethyl−2‐octene; estra−1,3,5(10)‐trien−17.beta.‐ol; ergosta−4,7,22‐trien3.beta‐ol	(Deepika et al., 2013)
08	*Syzygium campanulatum* Korth	*n*‐Hexane methanol	Leaf	(2S)−7‐Hydroxy−5‐methoxy−6,8‐dimethyl flavanone; (S)−5,7‐dihydroxy−6,8‐dimethyl‐flavanone; (E)−2ʹ,4ʹ‐ dihydroxy−6ʹ‐methoxy−3ʹ,5ʹ‐dimethylchalcone; betulinic and ursolic acids	(A. H. Memon et al., 2015)
09	*Syzygium calophyllifolium* Walp.	Ethyl acetate	Leaf	Squalene; γ‐eudesmol; βvatirenene; 4‐methoxy‐naphthalene−1‐carboxylic acid, Eicosane, α‐gurjunene, 9‐Eicosyne, Germacrene D, β‐Elemene, (‐)‐Isoledene	(Vignesh et al., 2013)
		Methanol	Fruit	3‐Piperidinamine, 1‐ethyl‐; N‐[3‐[n‐aziridyl]propylidene]−3‐methylaminopropylamine; and 1,3‐Propanediamine, n′‐[3‐(dimethylamino)‐n–n‐dimethyl	(Sathyanarayanan et al., 2018)
10	*Syzygium caryophyllatum* (L.) Alston	Hydrodistillation ethanol	Leaf(essential oil)	Phytol; α‐cadinol; globulol; humulene; and caryophyllene	(Khanh & Ban, 2020; Nadarajan & Pujari, 2014; Wathsara et al., 2020)
11	*Syzygium cordatum* Hochst ex C Krauss	Hydrodistillation	Leaf (EO)	Major compounds identified are 6,10,14‐trimethylpentadecane−2‐one; 2,3‐butanediol diacetate; n‐hexadeconic acid	(Chalannavar et al., 2011)
		_	Fruit	Major compounds identified are vanillic acid; caffeic acid; p‐coumaric acid; betulinic acid	(Maroyi, 2018)
		_	Bark	Major compounds identified are gallic acid; caffeic acid; arjunolic acid; epifriedelinol	(Maroyi, 2018)
12	*Syzygium corticosum* (Lour.)Merr.& L.M. Perry	Chloroform	Leaf	Major compounds identified are ursolic acid; fouquierol; melaleucic acid; 2,3‐dihydrosideroxylin	(Ren et al., 2018)
13	*Syzygium cumini* (L.) Skeels	_	Leaf	Major compounds identified are myricetin; myricetin−4‐methyl ether 3‐O‐α‐rhamnopyranoside; ellagic acid; caffeic acid; nilocetin; acylated flavonol glycosides; Beta‐sitosterol	(Chhikara et al., 2018)
		_	Fruit	Major compounds identified are raffinose; gallic acid; cyanidingdiglycoside; petunidin; delphinidin−3‐gentiobioside; malvidin−3‐laminaribioside	(Sowjanya et al., 2013)
		_	Bark	Major compounds identified are quercetin; kaemferol; 3,3‐di‐O‐methyl ellagic acid; friedelin	(Chhikara et al., 2018)
		_	Seed	Major compounds identified are quercetin; rutin; ferulic acids; corilagin	(Chhikara et al., 2018)
		_	Flower	Major compounds identified are kaemferol; Oleanolic acid; eugenol; erategolic acid	(Chhikara et al., 2018)
		_	Root	Major compounds identified are Isohamnetin−3‐O‐rutinside and flavonoid glycosides	(Chhikara et al., 2018)
		_	Pulp	Major compounds identified are myricetin deoxyhexoside; gallic acid; citronellol	(Chhikara et al., 2018)
14	*Syzygium densiflorum* Wall. ex Wt. &Arn.	Hydrodistillation	Leaf (EO)	Major constituents are β‐maaliene; isoledene;α‐gurjunene; β‐elemene; and β‐vatirenene	(Saranya et al., 2012)
15	*Syzygium fruticosum* DC.	Ethyl acetate	Leaf	Gallic acid methyl ester	(Nasrin et al., 2018)
16	*Syzygium formosum* (Wall.) Masam	Ethanol	Leaf	Main constituents are gallic acid; protocatechuic acid; ursolic acid; and quercetin	(Duyen Vu et al., 2019)
17	*Syzygium grande* (Wight) Walp.	Hydrodistillation	Leaf (EO)	Main constituents are β‐caryophyllene; sabinene; (E)‐β‐ocimene;α‐copaene	(Huong et al., 2017; Samy et al., 2014; Sarvesan et al., 2015)
		Hydrodistillation	Stem	Main constituents are β‐caryophyllene; sabinene; (E)‐β‐ocimene; δ‐Cadinene	(Huong et al., 2017)
18	*Syzygium guineense* (Willd.) DC.	n‐hexane	Leaf	Major compounds are tetratriacontane; 9‐octadecanoic acid; n‐hexadecenoic acid; and tetratriacontane	(Abok & Manulu, 2016)
19	*Syzygium jambos* L. (Alston)	Hydrodistillation	Leaf (EO)	Major compounds identified are (E)‐caryophyllene; n‐heneicosane; α‐humulene; thujopsan−2‐α‐ol	(Rezende et al., 2013)
		_	Stem bark	Major compounds identified are hexadecanoic acid; linoleic acid; and n‐butylidenephthalide	(LIN et al., 2013)
20	*Syzygium lineatum* (DC.) Merr. & L.M. Perry	Hydrodistillation	Leaf (EO)	Major compounds identified are β‐caryophyllene; α‐pinene; α‐selinene; and α‐humulene	(Khanh & Ban, 2020; Ruma, 2016)
21	*Syzygium lanceolatum* (Lam.) Wt. & Arn.	Hydrodistillation	Leaf (EO)	Major compounds identified are phenyl propanal; β‐caryophyllene; α‐humulene; and caryophyllene oxide. In another study, it was found that major compounds identified are 2,8‐Dimethyl−7‐methylene−1,8‐nonadien−3‐yne; germacrene D; elixene	(Benelli et al., 2018; Muthumperumal et al., 2016)
22	*Syzygium luehmannii* (F. Muell.) L.A.S. Johnson	Methanol & aqueous	Leaf	Major compounds identified are 2‐Ethyl−1‐hexanol; 2‐heptyl−1,3‐dioxolane; 1‐methyloctyl butyrate; Linalool; exo‐fenchol; 1‐terpineol; endo‐borneol; terpinen−4‐ol; and caryophyllene	(Noé et al., 2019)
23	*Syzygium legatii* Burtt Davy & Greenway	Acetone	Leaf	Major compounds identified are friedelan−3‐one; tetradecane; ethanedicarboxamide; dodecane	(Ibukun M Famuyide et al., 2020)
24	*Syzygium malaccense* (L.)Merr. & L.M. Perry	Hydrodistillation	Leaf	Major compounds identified are p‐cymene; (−)‐β‐caryophyllene; (−)‐β‐pinene and α‐terpineol	(Karioti et al., 2007)
25	*Syzygium myrtifolium* Walp.	Ethanol	Leaf	Major compounds identified are 1‐Octadecene; bis (2‐ethylhexyl) hexanedioic; and bis (2‐ethylhexyl) phthalate	(Novianti et al., 2019)
26	*Syzygium polyanthum* (Wight) Walp.	Aqueous	Leaf	Major compounds identified are 9‐octadecenoic; eicosanoic acid	(Widjajakusuma et al., 2019)
		n‐hexane	Leaf	Major compounds identified are squalene; phytol; α‐pinene; and α‐tocopherol	(Abd Rahim et al., 2018)
		Ethyl acetate	Leaf	Major compounds identified are squalene; phytol; β‐sitosterol	(Abd Rahim et al., 2018)
		Methanol	Leaf	Major compounds identified are squalene; β‐sitosterol; pyrogallol; phytol	(Abd Rahim et al., 2018)
27	*Syzygium paniculatum* Gaertn.	Hydrodistillation (VO)	Fruit	Major compounds identified are α‐pinene; (Z)‐β‐ocimene; limonene; and α‐ terpineol	(Quijano‐Célis et al., 2013)
		Hydrodistillation (VO)	Bark	Major compounds identified are α‐pinene; n‐hexadecanoic acid; limonene; and farnesol	
28	*Syzygium samarangense* (Blume) Merr. and L.M. Perry	Methanol	Leaf	Major compounds identified were (epi)‐catechin‐(epi)‐gallocatechin; (epi)‐gallocatechin gallate; (epi)‐catechin‐afzelechin; myricetin pentoside	(Sobeh et al., 2018)
		Hydrodistillation	Leaf (EO)	Major compounds identified were β‐selinene; α‐selinene; γ‐terpinene; β‐caryophyllene; and β‐gurjunene	(Reddy et al., 2011)
29	*Syzygium zeylanicum* (L.) DC.	Hydrodistillation	Leaf (EO)	Major compounds identified were α‐humulene and β‐elemene	(Govindarajan & Benelli, 2016)
		_	Seed	Major compounds identified were oleic acid; linoleic acid and palmitic acid	(Shilpa & Krishnakumar, 2015)
		_	Pulp	Major compounds identified were oleic acid; linoleic acid; and palmitic acid	(Shilpa & Krishnakumar, 2015)

### 4.2 Phenols

Phenol is an aromatic hydrocarbon compound having antioxidant, antimicrobial, anti‐inflammation activities (Ağagündüz et al., 2021; Hossen et al., 2021; Minatel et al., 2017). *S. alternifolium* is rich in phenols in which nearly 40 types of different compounds were identified in methanol extracts of stem barks, leaves, and fruits through GC‐MS analysis. Among these, seven compounds were phenols (eg1‐butanol, 2‐furanmethanol, propol, methylpropylcarbinol, flavone, 2’,5,6,6’‐tetramethoxy‐; Yugandhar & Savithramma, 2017). Three polyphenols (e.g. gallic acid, myricitrin, and quercitrin; Figure 1), isolated from the methanol extract of *S*. *antisepticum* leaves, showed strong antioxidant activity (Mangmool et al., 2021). Methanol extract of *S. aqueum* leaves contained a few phenolic compounds (e.g. caffeic acid; Sobeh, Mahmoud, et al., 2018, 2018). Aqueous extract of *S*. *aromaticum* seeds contained a phenolic compound, eugenol acetate (Ajiboye et al., 2016). *S. cordatum* fruit extracts contained several phenolic compounds determined by HPLC/TLC. Among these, methanol fruit extract contained vanillic acid, caffeic acid, and p‐coumaric acid (Maroyi, 2018). *S. cumini* contained very few phenolic compounds in various parts of the plants such as leaf extract (e.g. ellagic acid; caffeic acid), fruit extract (e.g. gallic acid; Sowjanya et al., 2013), bark extract (e.g. 3,3‐di‐O‐methyl ellagic acid; 3,3,4‐tri‐O‐methyl ellagic acid), seed extract (e.g. ferulic acid), and flower extract (e.g. oleanolic acid; eugenol; Chhikara et al., 2018). *S. formosum* ethanolic leaf extract contained 28 compounds. Among these, four compounds were phenolic compounds (e.g., gallic acid; protocatechuic acid) determined by HPLC method (Duyen Vu et al., 2019). Gallic acid, isolated from methanol stem extract of *S*. *litorale*, exhibited strong antioxidant activity against 2,2‐diphenyl‐1‐picrylhydrazyl (DPPH; Tukiran et al., 2016). *S. samarangense* methanol leaf extract also contained gallic acid and p‐coumaroylquinic acid (Martínez et al., 2020; Sobeh et al., 2018).

### 4.3 Tannins

Tannins are polyphenolic biomolecules that have antioxidant, antimicrobial, antinutritional, anticancer, cardioprotective properties (Babar et al., 2019; Smeriglio et al., 2017). Methanol extract of *S. aqueum* leaves contained few number of tannin compounds (e.g. galloylquinic acid; Sobeh, Mahmoud, et al., 2018, 2018). *S. cumini* contained very few tannin compounds in various parts of the plants such as leaf extract (e.g. nilocetin) and seed extract (e.g. corilagin; Chhikara et al., 2018). *S. samarangense* methanol leaf extracts also contained galloylquinic acid and quinic acid (Khan et al., 2020; Sobeh et al., 2018).

### 4.4 Terpenoids

Terpenoids belong to the class of organic compounds which have a hepatoprotective, anti‐inflammatory, antimicrobial, analgesic, and immunomodulatory activities (Akkol, Çankaya, et al., 2021; Dzubak et al., 2006; Reza et al., 2018). n‐Hexane methanol extract of *S. campanulatum* leaves contained betulinic and ursolic acids assessed in HPLC method (Bristy et al., 2020; Memon et al., 2015; Rahman et al., 2020). Ethyl acetate extract of leaves of *S. calophyllifolium* contained higher proportions of sesquiterpenoids and triterpenoid compounds that showed effective antimicrobial activity. The extract also showed strong cytotoxicity and antioxidant activity. The major constituent was squalene assessed through GC‐MS analysis (Vignesh et al., 2013). *S. cordatum* fruit, bark, and wood extracts contained several triterpenoid compounds determined by TLC, IR, MS, CC. Among them, fruit extract contained betulinic acid. Bark and wood extract contained arjunolic acid, epifriedelinol, and friedelin (Maroyi, 2018). Chloroform extract of *S*. *corticosum* leaves contained 19 compounds. Among these, seven compounds were triterpenoids, and the major triterpenoid compounds were ursolic acid and melaleucic acid, obtained through chromatographic separation (Ren et al., 2018). *S. cumini* contained very few triterpenoid compounds in various parts of the plants such as leaf extract (e.g. acylated flavonol glycosides) and bark extract (e.g. friedelin; Chhikara et al., 2018). *S. formosum* ethanolic leaf extract contained 28 compounds. Among these, 13 compounds were triterpenoids (e.g., maslinic acid; ursolic acid) determined by HPLC method (Duyen Vu et al., 2019). Dichloromethane/methanol (1:1) extract of *S*. *guineense* was reported to contain 2, 3, 23‐trihydroxy methyl oleanate (Abera et al., 2018). *S. legatii* acetone leaf extract yielded 15 compounds with a triterpenoid compound, friedelan‐3‐one, determined by GC‐MS analysis (Famuyide et al., 2020).

### 4.5 Alkaloids

Alkaloids are naturally occurring organic compounds, which consist of at least one nitrogen atom and also have a wide range of pharmacological activities such as antibacterial, anticancer, analgesic, antihyperglycemic, and antimalarial (Tareq et al., 2020; Uddin et al., 2018). *S*. *cumini* seeds are reported to contain alkaloid, jambosine, and showed antidiabetic effect (Ayyanar & Subash‐Babu, 2012). There are various compounds isolated from different parts of *Syzygium* species such as *S. cumini, S. polyanthum,* and *S. aromaticum* (Abd Rahim et al., 2018; Hasanuzzaman et al., 2016). Methanol extract of *S*. *cordatum* fruit pulp and seed contains alkaloids (Sidney et al., 2015a).

### 4.6 Glycosides

Glycosides are the compounds in which a sugar is bound to another functional group via a glycosidic bond and many plants preserve chemicals as inactive form of glycosides (Ali Reza et al., 2021). It has several pharmacological activities, including antiarrhythmic and antihyperglycemic. 2,4,6‐Trihydroxy‐3‐methylacetophenone‐2‐*O*‐*β*‐d‐glucoside, a new acetophenone, was isolated from the flower buds of *S*. *aromaticum* (Ryu et al., 2016). *S*. *cumini* seeds contained glycoside jambolin or antimellin and showed antidiabetic effect by inhibiting the diastatic conversion of starch into sugar (Ayyanar & Subash‐Babu, 2012). There are few compounds found in different parts of *S*. species such *S. cumini* and *S. polyanthum* (Abd Rahim et al., 2018; Hasanuzzaman et al., 2016; Kusuma et al., 2011).

### 4.7 Others

There are so many classes of compounds such as alkaloid, fatty acid, saponin, anthocyanin, glycosides, etc. Gas chromatography coupled with mass spectrometry (GC‐MS) analysis of *S. anisatum* leaf essential oil showed that it contained large amounts of phenylpropene compounds (e.g. E‐anethole, methyl chavicol) and also contained monoterpenoids (e.g. 1,8‐Cineole; Brophy & Boland, 1991). Methanol extract of *S. aqueum* leaves contained 87 different compounds which were determined through high‐resolution LC‐ESI‐MS/MS analysis; among them, major proanthocyanins were samarangenin A; (epi)‐gallocatechin gallate; (epi)‐catechin‐(epi)‐gallocatechin‐(epi)‐gallocatechin gallate; and major ellagitannins compound was digalloyl‐hexahydroxydiphenoyl (HHDP)‐hexoside (Sobeh, Mahmoud, et al., 2018, 2018). GLC‐MS analysis of leaf essential oil contained large amounts of sesquiterpenes (e.g., α‐selinene; β‐caryophyllene; and β‐selinene; Sobeh et al., 2016). Extract of *S. aromaticum* clove leaves contained major constituents of phenylpropanoid compounds (e.g. eugenol), sesquiterpenes (e.g. β‐caryophyllene), and ester (e.g. 3‐hexen‐1‐ol and hexyl acetate); clove bud oils contained phenylpropanoid compounds (e.g. eugenol); sesquiterpenes (e.g. β‐caryophyllene), founded through statistical analysis using GC‐MS (Kasai et al., 2016). Methanol extract of *S. arnottianum* leaves contained 11 phytochemical compounds, among them some major compounds are oxazole; ketone (e.g. cyclopentanone); 1,2,4,5‐tetraethyl‐2‐thiopheneacetic acid; hexyl ester hydrazine; and 4,5‐dihydro‐2‐methyl‐dichloro acetic acid, analyzed through GC‐MS analysis (Krishna & Mohan, 2012). Both methanol and aqueous extracts of *S. austral* leaves contained major compounds such as 1‐vinylheptanol; 2‐ethyl‐1‐hexanol; 2‐heptyl‐1,3‐dioxolane; 1‐methyloctyl butyrate; and several terpenoids (e.g. linalool; exo‐fenchol; and 1‐terpineol), assessed through GC‐MS analysis (Noé et al., 2019). Ethyl acetate extract of *S. benthamianum* leaves contained total 24 compounds; among them, major constituents are 4‐(4‐ethylcyclohexyl)‐1‐pentyl‐cyclohexene; linoleic acid(fatty acid); 2,6,10,14,18‐penta‐methyl‐2,6,10,14,18‐eicosapentaene; 9,17‐octadecadienal,(z)‐; Z,E‐3,13‐octadecadien‐1‐ol; and 7‐pentadecyne, assessed through GC‐MS analysis (Kiruthiga et al., 2011). Further, GC‐MS analysis of leaves essential oil showed that it contained a total of 63 compounds; the major compounds obtained were sitosteryl acetate; stigmastan‐3,5,22‐trien; 2,6‐dimethyl‐2‐octene; estra‐1,3,5(10)‐trien‐17.beta.‐ol; ergosta‐4,7,22‐trien3.beta‐ol; and a number of other minor compounds (Deepika et al., 2013). *S. campanulatum* n‐hexane and methanol leaf extract contained chalcone (e.g. (E)‐2ʹ,4ʹ‐dihydroxy‐6ʹ‐methoxy‐3ʹ,5ʹ‐dimethylchalcone), assessed in HPLC method (A. H. Memon et al., 2015). Ethyl acetate extract of *S. calophyllifolium* leaves contained 60 compounds; among these, major compounds are γ‐eudesmol; β‐vatirenene; 4‐methoxy‐naphthalene‐1‐carboxylic acid; α‐gurjunene; eicosane; germacrene D, assessed through GC‐MS analysis (Vignesh et al., 2013). Methanol extract of fruits contained 12 compounds; among these, major compounds are 3‐piperidinamine; 1‐ethyl‐; N‐[3‐[n‐aziridyl]propylidene]‐3‐methylaminopropylamine; 1,3‐propanediamine; n′‐[3‐(dimethylamino)‐n–n‐dimethyl, determined through GC‐MS analysis (Sathyanarayanan et al., 2018). *S. caryophyllatum* leaves yielded essential oil upon hydrodistillation and identified 58 compounds through GC‐MS analysis; the major compounds are phytol; α‐cadinol; globulol; humulene; and caryophyllene (Wathsara et al., 2020). *S. cordatum* leaves yielded essential oil upon hydrodistillation and identified 60 compounds through GC‐FID (Gas chromatography analysis) and GC‐MS analysis; the major compounds identified are 6,10,14‐trimethylpentadecane‐2‐one; 2,3‐butanediol diacetate; n‐hexadeconic acid (Chalannavar et al., 2011). **Mycaminose**, a carbohydrate isolated from *S*. *cumini* seed extract, exhibited antidiabetic effects against STZ‐induced diabetic rats (A. Kumar et al., 2013). *S. densiflorum* essential oil compositions were identified from hexane extract, a total of 84 compounds were identified among which β‐maaliene; isoledene; α‐gurjunene; β‐elemene; and β‐vatirenene, analyzed by GC‐MS analysis (Saranya et al., 2012). *S*. *grande* leaves and stem yielded essential oil upon hydrodistillation and identified 22 and 43 compounds, respectively. The compounds were determined by GC‐MS analysis. Among all the compounds, the major constituents of leaves are β‐caryophyllene; sabinene; (E)‐β‐ocimene; α‐copaene, and the major constituents of stem are β‐caryophyllene; sabinene; (E)‐β‐ocimene; δ‐Cadinene. In both of them, majority of the compounds are sesquiterpene and monoterpene hydrocarbons (Huong et al., 2017). *S. guineense* n‐hexane leaf extract contained 12 compounds identified by GC‐MS analysis, and major compounds among them were tetratriacontane; 9‐octadecanoic acid; n‐hexadecenoic acid; and tetratriacontane. Organic acid followed by hydrocarbon are the major classes of the identified compounds (Abok & Manulu, 2016). *S. jambos* leaf essential oil yielded 62 compounds in GC‐MS analysis. Among them, major compounds identified are (E)‐caryophyllene; n‐heneicosane; α‐humulene; thujopsan‐2‐α‐ol (Rezende et al., 2013). Its stem bark essential oil yielded 22 compounds in GC‐MS analysis. Among them, major compounds identified are hexadecanoic acid; linoleic acid; and n‐butylidenephthalide (LIN et al., 2013). *S. lineatum* leaves yielded essential oil upon hydrodistillation and identified compounds through GC‐MS and gas chromatography flame ionization detector (GC‐FID) analysis. Sesquiterpenes were the major class of compounds. Among them, major compounds identified are β‐caryophyllene; α‐pinene; α‐selinene; and α‐humulene (Khanh & Ban, 2020). *S. lanceolatum* leaves yielded essential oil upon hydrodistillation, and 18 compounds in one study and 106 compounds in another study were identified through GC‐MS analysis. Alkenes were the major class of compounds, followed by sesquiterpenes. Among them, major compounds identified were phenyl propanal; β‐caryophyllene; α‐humulene; caryophyllene oxide; 2,8‐dimethyl‐7‐methylene‐1,8‐nonadien‐3‐yne; and germacrene D (Benelli et al., 2018; Muthumperumal et al., 2016). Both methanol and aqueous extracts of *S. luehmannii* leaves contained major compounds such as 2‐ethyl‐1‐hexanol; 2‐heptyl‐1,3‐dioxolane; 1‐methyloctyl butyrate; several terpenoids (e.g. Linalool; exo‐fenchol; 1‐terpineol; endo‐borneol; terpinen‐4‐ol; and caryophyllene), assessed through GC‐MS analysis (Noé et al., 2019). *S. legatii* acetone leaf extract yielded 15 compounds; among them, major compounds identified were friedelan‐3‐one; tetradecane; ethanedicarboxamide; dodecane, determined by GC‐MS analysis (Ibukun M Famuyide et al., 2020). *S. malaccense* leaves yielded essential oil upon hydrodistillation and identified 38 compounds through GC‐MS analysis. Monoterpenes (e.g. p‐cymene; (−)‐β‐pinene; α‐terpineol; (+)‐α‐pinene) were the major class of compounds, followed by sesquiterpene (e.g. (−)‐β‐caryophyllene; Karioti et al., 2007). *S. myrtifolium* ethanol leaf extracts yielded few compounds; among them, major compounds identified were 1‐octadecene; bis (2‐ethylhexyl) hexanedioic and bis (2‐ethylhexyl) phthalate, determined by GC‐MS analysis (Novianti et al., 2019). *S*. *polyanthum* aqueous leaf extract exhibited 12 compounds in GC‐MS analysis. Methyl esters were the major class of compounds. The major compounds are 9‐octadecenoic and eicosanoic acid (Widjajakusuma et al., 2019). *S. paniculatum* fruits yielded volatile oil upon hydrodistillation and identified 155 compounds through GC‐FID (Gas chromatography analysis) and GC‐MS analysis; the major compounds identified were α‐pinene; (Z)‐β‐ocimene; limonene; and α‐ terpineol, rich in terpenes (Quijano‐Célis et al., 2013). Moreover, several leaf extract of *S. paniculatum* are abundant in squalene; β‐sitosterol; phytol (Abd Rahim et al., 2018). The major compounds of bark were α‐pinene; n‐hexadecanoic acid; limonene; and farnesol (Okoh et al., 2019). *S. samarangense* leaves yielded essential oil upon hydrodistillation and identified 14 compounds through GC‐MS analysis; the major compounds identified were β‐selinene; α‐selinene; γ‐terpinene; β‐caryophyllene; and β‐gurjunene (Reddy & Jose, 2011). *S. zeylanicum* leaves yielded essential oil upon hydrodistillation and identified 18 compounds through GC‐MS analysis; the major compounds identified were α‐humulene and β‐elemene (Govindarajan & Benelli, 2016). Fatty acid composition of seeds and pulp yielded few compounds; among them, the major compounds were oleic acid; linoleic acid; and palmitic acid, same for both parts of the plant, determined by GC‐MS analysis (Shilpa & Krishnakumar, 2015).

## 5 PHARMACOLOGICAL ACTIVITIES

### 5.1 Antioxidant activities

**FIGURE 2:**
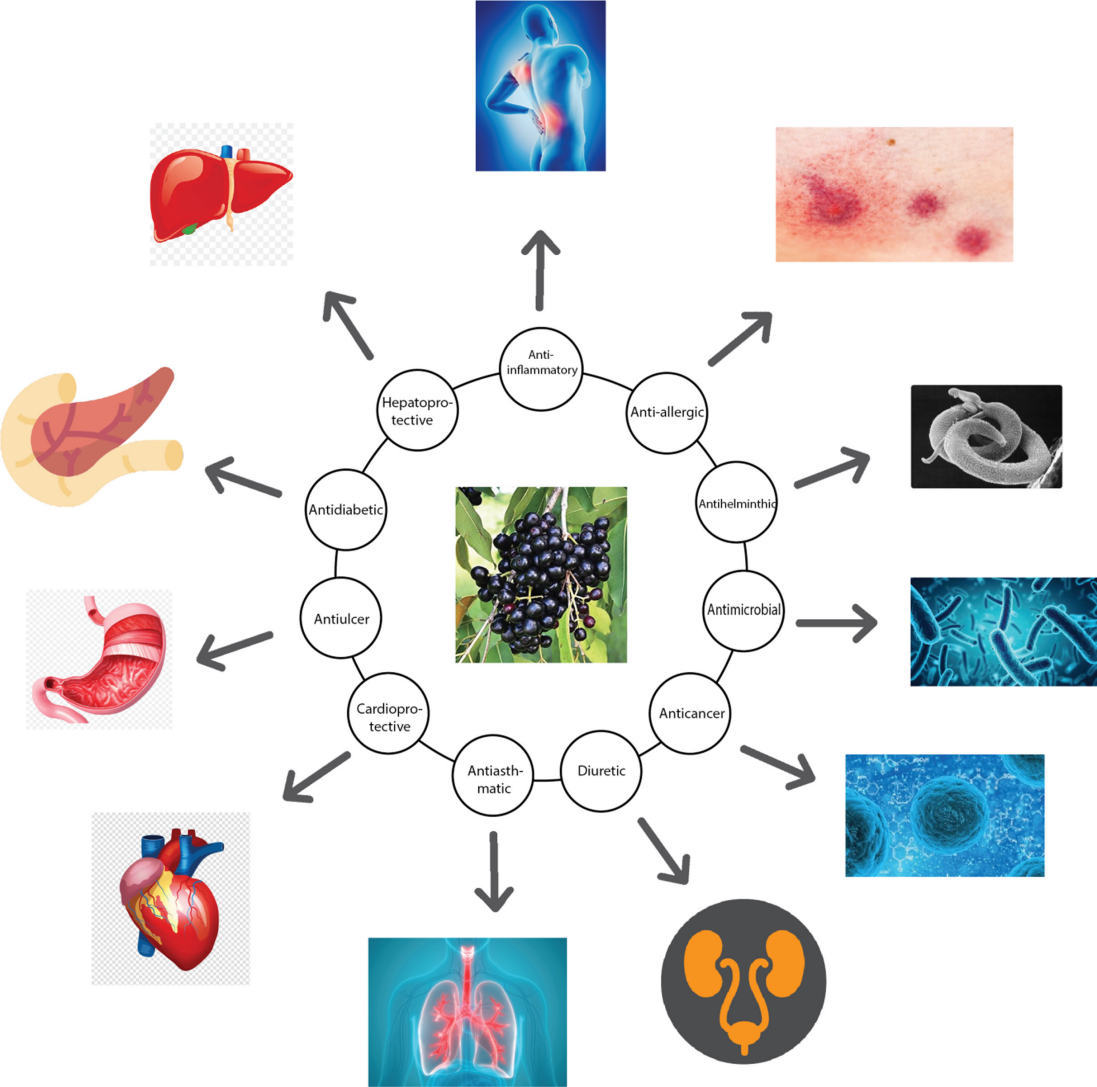
Graphical representation of pharmacological activities of different species of *Syzygium* genus

Antioxidants are the elements which scavenge free radicals, improve protection level from oxidative damage, and also help in decreasing or inhibiting oxidative stress (OS; Figure 2). Many compounds isolated from plants are considered to be natural resources of antioxidants (Table 3). Consumption of several foods which are rich in flavonoid and phenolic compounds exhibits antioxidant effects that can be advantageous for health (Majumder et al., 2017). The ability to scavenge the oxygen free radicals was displayed by the leaf of *S. anisatum* (Konczak et al., 2010). *S. aqueum* leaf extract showed strong antioxidant properties in vitro and protected human keratinocytes (HaCaT cells) against UVA damage (Sobeh, Mahmoud, et al., 2018, 2018). Water and methanol extracts of *S. aromaticum* clove buds and leaves exhibited effective antioxidant activity (Kasai et al., 2016). Its essential clove oil showed high DPPH radical scavenging capacity and low hydroxyl radical inhibition (Radünz et al., 2019). Ethyl acetate leaf extract of *S. benthamianum* was found to act as potent free radical scavengers in comparison with BHT, a commercial antioxidant. Moreover, a concentration of 400 μg/ml of the extract showed significant inhibition of DPPH radical scavenging activity (Kiruthiga et al., 2011). Ethyl acetate extract of *S. calophyllifolium* leaves showed higher radical scavenging activity against DPPH free radical, and the activity of was concentration‐dependent manner (Vignesh et al., 2013). Its methanol extract of fruits also showed antioxidant activity (Sathyanarayanan et al., 2018). Ethyl acetate fraction of *S. caryophyllatum* leaves and n‐hexane fraction of *S. caryophyllatum* fruits exhibited significant antioxidant activity (Wathsara et al., 2020). Methanol extract of *S. cordatum* plant was found to be more effective in scavenging DPPH free radicals and exhibited antioxidant activity (Mzindle, 2017). *S. cumini* methanol leaf extract exhibited antioxidant activity using the DPPH free radical scavenging and ferric‐reducing antioxidant power (FRAP) assays (Ruan et al., 2008). Its seed powder with high carbohydrate diet supplementation prevented the rise of plasma OS markers (superoxide dismutase, catalase, and glutathione) and restored the anti‐oxidative enzymes activity (Ulla et al., 2017). Moreover, fruit extract of *S. cumini* showed antioxidant activity (Singh et al., 2016). Ethanol extract of *S. densiflorum* leaves showed significant antioxidant activity by decreasing super oxide dismutase (SOD) and TBARS levels at a dose of 200 mg/kg (MK et al., 2013). Ethanol extract of fruit also showed antioxidant activity by reducing blood glucose level (Krishnasamy et al., 2016). *S. fruticosum* chloroform fraction of methanol bark extract showed the highest free radical scavenging activity with IC_50_ value of 20.01 µg/ml (Chadni et al., 2014). Its methanol seed extract also exhibited significant antioxidant activity (S. Islam et al., 2013). Ethanol extract of *S. formosum* leaves showed better DPPH‐scavenging activities (Lee et al., 2006). *S. grande* leaves essential oil and leaf aqueous extract showed higher scavenging activity against hydrogen peroxide and in peritoneal macrophages of rat by dihydrofluorescein assay (Jothiramshekar et al., 2014; Kukongviriyapan et al., 2007). *S. gratum* aqueous and ethanol leaf extracts exhibited strong antioxidant and intracellular oxygen radical scavenging activities. Its aqueous leaf extract was further examined in C57BL/6J mice and showed antioxidant activity along with cytoprotective effect (Senggunprai et al., 2010). *S. guineense* ethanol leaf extract exhibited antioxidant activity against ferric nitriloacetate‐induced stress in the liver, heart, kidney, and brain tissues of Wistar rat homogenates by inhibiting the lipid peroxidation and restored the enzymatic and nonenzymatic activities (Nzufo et al., 2017). Ethanol leaf extract of *S. jambos* exhibited significant antioxidant activity with 50% inhibitory concentration (Bonfanti et al., 2013; H. Hossain et al., 2016; Sharma et al., 2013). Its fruit extract also exhibited antioxidant activity (Li et al., 2015). *S. luzonense* ethanol bark extract exhibited antioxidant activity (Walean et al., 2020). *S. lanceolatum* leaf essential oil exhibited strongest antioxidant activity with 69.97% inhibitory concentration, determined by the DPPH assay (Muthumperumal et al., 2016). *S. malaccense* methanol leaf extract exhibited strong antioxidant activity with 78.73% inhibitory concentration at a dose of 100μg /ml (Savitha et al., 2011). Its fruit extract also exhibited antioxidant activity (Nunes et al., 2016). *S. mundagam* methanol bark and leaf extracts exhibited antioxidant activity (Chandran et al., 2017). *S. maire* methanol fruit extract showed antioxidant activity (Gould et al., 2006). *S*. *polyanthus m*ethanol fruit extract exhibited antioxidant activity, determined by DPPH assay (Kusuma et al., 2011). *S*. *paniculatum* aqueous fruit extract exhibited antioxidant activity and decreased the levels of OS marker and protected the tissues (liver and kidney) against the cytotoxic action and OS‐induced diabetic rats (Konda et al., 2019; Vuong et al., 2014). *S. samarangense* methanol leaf extract showed antioxidant activity in Wistar rats by increasing the inhibition of GSH (reduced glutathione) and SOD levels and by decreasing the lipid peroxidation, determined by DPPH assay and reducing power assay (Majumder et al., 2017). *S. zeylanicum* methanol leaf extract exhibited strong antioxidant activity in DPPH assay (Nomi et al., 2012). Its fruit extract also exhibited antioxidant activity (Shilpa & Krishnakumar, 2015). Gallic acid methyl ester isolated and characterized from the ethyl acetate fraction of *S. fruticosum* leaves showed strong higher ferrous reducing antioxidant and DPPH free radical scavenging activities (Nasrin et al., 2018).

**TABLE 3:** A list of plants belonging to the *Syzygium* genus including plant parts with their pharmacological activities

Plant Name	Parts of Plant	Pharmacological activities	Reference
*Syzygium alternifolium* (WT.)* *WALP	Stem bark	Antimicrobial activity	(Yugandhar et al., 2015)
	Leaf	Anticancer activity (in vitro)	(Komuraiah et al., 2014)
	Seeds	Antihyperglycemic and antihyperlipidemic activities	(Kasetti et al., 2010)
*Syzygium anisatum* (Vicfkery) Craven & Biffen	Leaf	Antioxidant, antibacterial, anti‐inflammatory (in vitro), cytoprotective, and proapoptotic activities	(Bryant & Cock, 2016; Guo et al., 2014; Konczak et al., 2010; Sakulnarmrat et al., 2013)
*Syzygium aqueum* (Burm.f.) Alston	Leaf	Antioxidant, hepatoprotective, pain‐killing, anti‐inflammatory (in vitro), and antidiabetic activities	(T. Manaharan et al., 2013; Sobeh, Mahmoud, et al., 2018, 2018)
*Syzygium aromaticum* (L) Merr. & Perry.	Leaf	Antioxidant, antibacterial, and antibiofilm activities	(Kasai et al., 2016; Zhang et al., 2017)
	Seeds	Antibacterial activity	(Ajiboye et al., 2016)
	Clove	Antifungal, antimicrobial, antioxidant, anticandidal, hepatoprotective (in vitro), larvicidal, ovicidal potentiality, and anticancer activities	(Hina et al., 2017; Hong et al., 2018; Nirmala et al., 2019; Park et al., 2007; Radünz et al., 2019)
	Flower bud	Antiulcer, antioxidant (in vitro), anti‐inflammatory, antituberculosis, antidiabetic, and anthelmintic activities	(Chniguir et al., 2019; Kasai et al., 2016; Kaur & Kaur, 2015; Patil et al., 2014; Santin et al., 2011; Tahir et al., 2016)
*Syzygium australe* (H.L. Wendl. Ex Link) B. Hyland	Leaf	Antifungal activity (in vitro)	(Noé et al., 2019)
	Fruit	Antibacterial and antiproliferative activities	(Jamieson et al., 2014; Sautron & Cock, 2014)
*Syzygium benthamianum* (Wight ex Duthie) Gamble	Leaf	Antimicrobial, antioxidant, and anticancer activities	(Kiruthiga et al., 2011)
*Syzygium campanulatum* Korth	Leaf	Antiproliferative, antiangiogenesis, and antitumor activities	(Aisha et al., 2013; A. H. Memon et al., 2015)
*Syzygium calophyllifolium* Walp.	Bark	Antidiabetic, cytotoxic, analgesic, and anti‐inflammatory activities	(Chandran et al., 2016, 2018)
	Leaf	Antibacterial, antifungal, antioxidant, and anticancer activities (in vitro)	(Vignesh et al., 2013)
	Fruit	Antioxidant and antibacterial activities (in vitro)	(Sathyanarayanan et al., 2018)
*Syzygium caryophyllatum* (L.) Alston	Leaf	Antioxidant, antidiabetic (in vitro), antibacterial, antifungal, and anticancer activities	(Annadurai et al., 2012; Nadarajan & Pujari, 2014; Wathsara et al., 2020)
	Fruit	Antioxidant and antidiabetic activities (in vitro)	(Wathsara et al., 2020)
	Root	Anti‐inflammatory activity (in vitro)	(Heendeniya et al., 2018)
*Syzygium cordatum* Hochst ex C Krauss	Leaf	Antibacterial, antifungal, anti‐inflammatory activity; antidiarrheal, antidiabetic, antioxidant, antileishmanial, and antiplasmodial activities	(Bapela et al., 2017; I. E. Cock & van Vuuren, 2014; Deliwe & Amabeoku, 2013; Mulaudzi et al., 2012; Mzindle, 2017; Nondo et al., 2015)
	Fruit	Antibacterial and antidiarrheal activities	(Maliehe et al., 2015; Sidney et al., 2015b)
	Seed	Antibacterial and antidiarrheal activities	(Maliehe et al., 2015; Sidney et al., 2015b)
	Bark	Antibacterial, antifungal, antimutagenic, and antiplasmodial activities	(I. E. Cock & van Vuuren, 2014; Nciki et al., 2016; Nondo et al., 2015; Verschaeve et al., 2004)
*Syzygium corticosum* (Lour.)Merr.& L.M. Perry	Leaf	Anticancer activity (in vitro)	(Ren et al., 2018)
*Syzygium cumini* (L.) Skeels	Leaf	Antidiabetic, antioxidant, antinociceptive, anti‐leishmania, and antiallergic activities	(Brito et al., 2007; Quintans et al., 2014; Rodrigues et al., 2015; Ruan et al., 2008; Schoenfelder et al., 2010)
	Fruit	Antioxidant (in vitro), antibacterial, and anticancer activities	(Afify et al., 2011; Singh et al., 2016)
	Bark	Antihelmintic activity	(Kavitha et al., 2011)
	Seed	Antibacterial, antihyperlipidemia, antioxidant, antidiabetic, and anti‐arthritis activities	(A. Kumar et al., 2013; E. Kumar et al., 2008; Ulla et al., 2017; Yadav et al., 2017)
*Syzygium densiflorum* Wall. ex Wt. & Arn.	Leaf	Antidiabetic, antioxidant, antibacterial, and antifungal activities	(Eganathan et al., 2012; MK et al., 2013)
	Fruit	Antidiabetic, antihyperlipidemic, and antioxidant activities	(Krishnasamy et al., 2016)
*Syzygium fruticosum* (Roxb.) DC.	Leaf	Cytotoxic and thrombolytic activities	(Chadni et al., 2014)
	Bark	Antibacterial and antioxidant activities	(Chadni et al., 2014)
	Seed	Antioxidant and anticancer activities	(S. Islam et al., 2013)
*Syzygium formosum* (Wall.) Masam	Leaf	Antiallergic, anti‐inflammatory, and antioxidant activities	(Lee et al., 2006; Nguyen et al., 2018)
*Syzygium francisii* (F.M. Bailey) L.A.S. Johnson	Leaf	Antibacterial activity	(Ian Cock et al., 2013)
*Syzygium forte* (F. Muell.) B. Hyland	Leaf	Antibacterial activity	(Ian Cock et al., 2013)
*Syzygium grande* (Wight) Walp.	Leaf	Antibacterial and antioxidant activities	(Jothiramshekar et al., 2014; Sarvesan et al., 2015)
	Bark	Antidiabetic activity	(Myint, 2017)
*Syzygium gratum* (Wight) S.N. Mitra	Leaf	Antioxidant, cytoprotective, and anticancer activity	(Kukongviriyapan et al., 2007; Rocchetti et al., 2019; Senggunprai et al., 2010; Stewart et al., 2013)
*Syzygium guineense* (Willd.) DC.	Leaf	Anti‐inflammatory, analgesic, antibacterial, antimalarial, antidiarrheal, antidiabetic (in vitro), antioxidant, antihypertensive (in vivo), and vasodepressor (in vitro) activities	(Ayele et al., 2010; Djoukeng et al., 2005; Ezenyi & Igoli, 2018; Ezuruike et al., 2019; IOR et al., 2012; Nzufo et al., 2017; Tadesse & Wubneh, 2017)
	Fruit	Cytotoxicity and antihelmintic activities	(Maregesi et al., 2016)
	Stem bark	Antituberculosis and antispasmodic activities	(Malele et al., 1997; Oladosu et al., 2017)
	Whole plant	Anticancer activity	(Koval et al., 2018)
*Syzygium jambos* L. (Alston)	Leaf	Antidiabetic, antibacterial, anti‐inflammatory, antioxidant, hepatoprotective, antifungal (in vitro), antinociceptive, analgesic, antiulcerogenic, and anticancer activities	(Avila‐Peña et al., 2007; Bonfanti et al., 2013; Chua et al., 2019; Donatini et al., 2009; Gavillán‐Suárez et al., 2015; H. Hossain et al., 2016; M. R. Islam et al., 2012; Noé et al., 2019; Sharma et al., 2013)
	Fruit	Antitumor, cytotoxic, and antioxidant activities	(Li et al., 2015; Tamiello et al., 2018)
	Stem bark	Antibacterial (in vitro), antidiabetic, and antileukemic activities	(Djipa et al., 2000; Hettiarachchi et al., 2004; Pardede et al., 2020)
*Syzygium johnsonii*(F. Muell.) B. Hyland	Stem bark	Cytotoxicity and antibacterial activities	(Harris et al., 2011; Setzer et al., 2001)
*Syzygium luzonense* (Merr.)Merr.	Stem bark	Antibacterial, antioxidant, and antihyperglycemic	(Walean et al., 2020)
*Syzygium lineatum*(DC.)Merr.& L.M. Perry	Leaf	Cytotoxicity, anticancer, and antiproliferative activities	(Castillo et al., 2017; A; Castillo et al., 2018; Chua et al., 2019)
*Syzygium lanceolatum* (Lam.) Wt. & Arn.	Leaf	Larvicidal, antioxidant and antibacterial activities	(Benelli et al., 2018; Karuppusamy & Rajasekaran, 2009; Muthumperumal et al., 2016)
*Syzygium luehmannii* (F. Muell.) L.A.S. Johnson	Leaf	Antifungal activity (in vitro)	(Noé et al., 2019)
	Fruit	Antifungal (in vitro), antibacterial, and antiproliferative activities	(Jamieson et al., 2014; Noé et al., 2019; Sautron & Cock, 2014)
*Syzygium legatii* Burtt Davy & Greenway	Leaf	Antibacterial, antibiofilm, and anti‐quorum sensing activities	(I. Famuyide et al., 2019; I. M. Famuyide et al., 2019)
*Syzygium malaccense* (L.)Merr. & L.M. Perry	Leaf	Antifungal, antibacterial (in vitro), anti‐inflammatory, antioxidant, and cytotoxic (in vitro) activities	(Dunstan et al., 1997; Itam & Anna, 2020; Locher et al., 1995; Savitha et al., 2011)
	Fruit	Antioxidant activity	(Nunes et al., 2016)
	Bark	Antiviral activity (in vitro)	(Locher et al., 1995)
*Syzygium myrtifolium* Walp.	Leaf	Antidermatophytic, fungicidal, cytotoxic, antidiarrheal, and antispasmodic activities	(Abdul Hakeem Memon et al., 2020; Sit et al., 2018)
*Syzygium mundagam* (Bourd.) Chitra	Leaf	Antioxidant activity;	(Chandran et al., 2017)
	Bark	Antidiabetic, antioxidant, antiproliferative, analgesic, and anti‐inflammatory activities	(Chandran et al., 2017a; Chandran et al., 2017b, 2017c, 2017d, 2020)
*Syzygium maire* (A. Cunn) Sykes & Garn.‐Jones	Fruit	Antioxidant activity	(Gould et al., 2006)
*Syzygium moorei* F. Muell.	Leaf	Antibacterial and antihyperglycemic activities	(Ian Cock et al., 2013)
*Syzygium paniculatum* Gaertn.	Fruit	Antioxidant, anticancer (in vitro), antihyperglycemic, antihyperlipidemic, and antioxidant activities	(Konda et al., 2019; Vuong et al., 2014)
	Leaf	Anticancer (Cytotoxic) activity;	(Rocchetti et al., 2019)
*Syzygium polyanthum* (Wight) Walp.	Leaf	Antidiabetic, antibacterial, and anticancer activities	(Nordin et al., 2019; Ramli et al., 2017; Widjajakusuma et al., 2019; Widyawati et al., 2015)
	Fruit	Antioxidant activity	(Kusuma et al., 2011)
*Syzygium puberulum* Merr.& L.M. Perry	Leaf	Antibacterial activity	(Ian Cock et al., 2013)
*Syzygium stocksii* (Duthie) Gamble	Leaf	Antibacterial and antifungal activities	(Eganathan et al., 2012)
*Syzygium samarangense* (Blume) Merr. and L.M. Perry	Leaf	Antioxidant, hepatoprotective, antidiabetic, anti‐inflammatory, analgesic, CNS depressant, antibacterial, antidiarrheal, and anti‐obesity activities	(Adesegun et al., 2013; Ghayur et al., 2006; Kim et al., 2012; Majumder et al., 2017; Mollika et al., 2014; Qowiyyah et al.; Reddy et al., 2011; Resurreccion‐Magno et al., 2005; Sobeh et al., 2018)
	Fruit	Anticancer, antihyperglycemic, anti‐inflammatory, and antiapoptotic activities	(Khamchan et al., 2018; Shen & Chang, 2013; Shen et al., 2012; Simirgiotis et al., 2008)
	Bark	Anthelmintic and anti‐acne activities	(Gayen et al., 2016; Sekar et al., 2017)
*Syzygium wilsonii* (F. Muell.) B. Hyland	Leaf	Antibacterial activity	(Ian Cock et al., 2013)
*Syzygium zeylanicum* (L.) DC.	Leaf	Antioxidant, anti‐inflammatory, antibacterial, and larvicidal activities	(Anoop et al., 2015; Govindarajan & Benelli, 2016; Nomi et al., 2012; Shilpa et al., 2014)
	Fruit	Antibacterial and antioxidant activities	(Shilpa & Krishnakumar, 2015)

### 5.2 Anti‐inflammatory activity

Anti‐inflammatory is an action of a substance, which affects the CNS to block the pain signaling to the brain and helps to reduce inflammation and pain. Various compounds of several classes isolated from different species of the genus *Syzygium* exhibited anti‐inflammatory activity (Table 3). For instance, polyphenolic compounds help to reduce inflammation and also reduce pain. *S. anisatum* extract which is rich in polyphenols with the murine macrophage cells applied in various concentration inhibited the protein expression levels of iNOS and cyclooxygenase‐2 (COX‐2) in LPS‐activated RAW 264.7 cells and exhibited in vitro potential anti‐inflammatory activity (Guo et al., 2014). The polyphenol‐enriched leaf extract of *S. aqueum* exhibited promising anti‐inflammatory activities in vitro where it inhibited LOX, COX‐1, and COX‐2 with a higher COX‐2 selectivity than that of standards indomethacin and diclofenac and reduced the extent of lysis of erythrocytes upon incubation with hypotonic buffer solution (Sobeh, Mahmoud, et al., 2018, 2018). Aqueous extract of *S. aromaticum* flower buds inhibited human neutrophils myeloperoxidase and protected mice from LPS‐induced lung inflammation (Chniguir et al., 2019). *S. calophyllifolium* methanol bark extract at a dose of 200 mg/kg was found to be very effective against granuloma formation with an inhibition of 70.46% compared to standard drug indomethacin (57.81%), suggesting the efficiency of bark extract to inhibit the migration inflammatory cells and to prevent abnormal permeability of the blood capillaries and showed anti‐inflammatory activity (Chandran et al., 2018). *S. caryophyllatum* aqueous root extract exhibited concentration‐dependent anti‐inflammatory activity in vitro with an IC50 value of 6.229 μg/ml (Heendeniya et al., 2018). *S. cordatum* petroleum ether and dichloromethane extracts of leaves exhibited high inhibition activity toward both COX‐1 and COX‐2 (>70%; Mulaudzi et al., 2012). *S. formosum* ethanol leaf extract exhibited significant improvement in the inflammatory lesion in the small intestine and reduced the number of mast cells and eosinophils recruited to the lesion (Nguyen et al., 2018). *S. guineense* ethanol leaf extract exhibited significant (*p* < .05) anti‐inflammatory and analgesic effects on the writhing test at a concentration of 1000 mg/kg in rat models (IOR et al., 2012). *S. jambos* ethanol leaf extract exhibited anti‐inflammatory activity against the pathogenic *Propionibacterium acnes* through preventing the release of inflammatory cytokines IL‐8 and tumor necrosis factor‐alpha (TNF‐α) by suppressing them by 74%–99% (H. Hossain et al., 2016; Sharma et al., 2013). *S. malaccense* leaf extract exhibited anti‐inflammatory activity through inhibiting COX‐1 catalyzed prostaglandin biosynthesis (Dunstan et al., 1997). *S. mundagam* methanol bark extract exhibited anti‐inflammatory and analgesic activities by reducing inflammation and pain, respectively (Chandran et al., 2020). *S. samarangense* fruit extract exhibited anti‐inflammatory activity through preventing the intracellular inflammatory signal, reestablishing the PI3K‐Akt/PKB insulin signaling pathway and also increasing glucose uptake in TNF‐α treated FL83B mouse (Shen et al., 2012). Methanol leaf extract also exhibited anti‐inflammatory activity through significant (*p* < .05) inhibition of carrageenan‐induced paw edema (Mollika et al., 2014). *S. zeylanicum* ethyl acetate leaf extract exhibited anti‐inflammatory activity through inhibition of cyclooxygenase, 5‐lipoxygenase, and also protein denaturation (Anoop, Bindu, & Review, 2015).

### 5.3 Antibacterial activities

Antibacterial agents are the substances used principally against pathogenic bacteria to kill or inhibit them to protect cells. Aqueous extract of *S. alternifolium* stem bark showed antimicrobial properties (Yugandhar et al., 2015). *S. anisatum* methanol and aqueous leaf extract significantly inhibited both gram‐positive and gram‐negative bacteria (Bryant & Cock, 2016). *S. aromaticum* leaf essential oil eugenol exhibited antibacterial activity (90.84%) against *P*. *gingivalis* concentration of 31.25 μM (Zhang et al., 2017). *S*. *aromaticum* seed extract showed antibacterial activity with minimum inhibitory concentration (MIC) and minimum bactericidal concentration (MBC) values of 0.06 and 0.10 mg/ml, respectively. Time kill susceptibility at MBC value showed significant decrease in the optical density and colony‐forming unit (CFU) of *Escherichia coli*, *Pseudomonas aeruginosa,* and *Staphylococcus aureus* (Ajiboye et al., 2016). *S. aromaticum* essential oil showed in vitro inhibitory and bactericidal effects against *Staph. aureus* (Radünz et al., 2019). Methanol extract of *S. austral* fruit exhibited greater antibacterial activity in disk diffusion assay method (Sautron & Cock, 2014). Ethyl acetate extract of *S. benthamianum* leaves showed antibacterial activity by inhibiting the growth of *Staph*. *aureus* at the MIC of 500 μg/ml, and other microbial species used in this study showed MIC at 250 μg/ml (Kiruthiga et al., 2011). Ethyl acetate extract of *S. calophyllifolium* leaves showed maximum zone of inhibition against *E. Faecalis* and expressed its antibacterial activity (Vignesh et al., 2013). Its methanol extract showed antibacterial activity against *E*. *coli* and others at a concentration of 100 mg/ml (Sathyanarayanan et al., 2018). Isolated compounds from *S. caryophyllatum* leaf essential oil exhibited potent antibacterial activity against both gram‐positive and gram‐negative bacteria carried out by disk diffusion method against six pathogenic bacteria (e.g. *Bacillus cereus*; Nadarajan & Pujari, 2014). Aqueous extract of *S. cordatum* leaves exhibited good antibacterial activity, determined by using the microdilution bioassay in 96‐well plate (Mulaudzi et al., 2012). Its methanol extract of fruits and seeds also showed antibacterial activity. The fruit pulp extract exhibited the lowest MIC value of 3.13 mg/ml against some gram‐positive and gram‐negative bacteria, while the seed extract showed the lowest MIC (Maliehe et al., 2015; Sidney et al., 2015b). The aqueous and dichloromethane–methanol extracts of barks exhibited antibacterial activity by producing inhibition effect against several bacterial pathogens, determined by the microtiter plate dilution assay (Nciki et al., 2016). *S. cumini* methanol seed extract exhibited antibacterial activity against the *B. subtilis* by agar well diffusion assay. The data showed that *B. subtilis* is susceptible to methanol seed extract with a zone of inhibition of 20.03 mm (Yadav et al., 2017). Its fruit extract also showed antibacterial activity (Singh et al., 2016). Ethyl acetate extract of *S. densiflorum* leaves exhibited antibacterial activity against six bacterial strains (e.g. *Pseudomonas aeroginosa*), determined by disk diffusion method (Eganathan et al., 2012). Chloroform and aqueous fractions of methanol bark extract of *S. fruticosum* showed mild antibacterial activity against both gram‐positive (e.g. *B*. *cereus*) and gram‐negative bacteria (e.g. *E*. *coli*) with zone of inhibition ranging from 7 to 14 mm as compared to standard ciprofloxacin (zone of inhibition of 50 mm), determined by disk diffusion method (Chadni et al., 2014). *S. francisii, S. forte, S. moorei, S. puberulum,* and *S. wilsonii* leaf methanol extracts inhibited the growth of several gram‐positive and gram‐negative bacterial strains and exhibited antibacterial activity. Moreover, leaf extract of these species showed lower toxicity (LC50 > 1000 μg/ml)) except *S. forte* (Ian Cock et al., 2013). *S*. *grande* leaf essential oil exhibited the antibacterial activity and produced a maximum zone of inhibition against *B*. *subtilis* and a minimum zone of inhibition against *E*. *coli* (Sarvesan et al., 2015). *S. guineense* leaf extract showed antibacterial activity against gram‐positive and gram‐negative bacteria, determined by disk diffusion method (Djoukeng et al., 2005). *S. jambos* ethanol leaf extract exhibited antibacterial activity by inhibiting the growth of *Propionibacterium acnes* with an MIC value of 31.3 and 7.9 μg/ml (Sharma et al., 2013). Acetone and aqueous bark extracts also exhibited antibacterial activity against several bacterial strains, determined by the agar dilution method (Djipa et al., 2000). *S. johnsonii* ethanol and chloroform bark extracts exhibited antibacterial activity against both gram‐positive bacteria (e.g. *B*. *cereus*), with MIC value 624 and 1250 ppm, respectively, and gram‐negative bacteria (e.g. *P*. *aeruginosa*), with MIC value 156 and 624 ppm, respectively, determined using the microbroth dilution technique (Harris et al., 2011; Setzer et al., 2001). *S. luzonense* ethanol bark extract exhibited antibacterial activity against both gram‐positive bacteria (e.g., *Staph*. *aureus*) and gram‐negative bacteria (e.g. *E*. *coli*), determined by Kirby–Bauer method (Walean et al., 2020). *S. lanceolatum* leaf essential oil exhibited antibacterial activity against six bacterial strains, namely, *B. cereus*, *B. licheniformis*, *Staph*. *aureus*, *Staph*. *hominis*, *A. viridian* and *E. coli* (Muthumperumal et al., 2016). *S. luehmannii* methanol fruit extracts exhibited greater antibacterial activity in disk diffusion assay (Sautron & Cock, 2014). *S. legatii* acetone leaf extract showed strong antibacterial activity against both gram‐positive (e.g., *B*. *cereus*) and gram‐negative bacteria (e.g., *E*. *coli*) with zone of significant inhibition (I. M. Famuyide et al., 2019). *S. malaccense* aqueous leaf extract exhibited antibacterial activity against only gram‐positive bacteria (e.g., *S. pyogenes*, *Staph*. *aureus*) with significant zone of inhibition (Locher et al., 1995). *S*. *polyanthus m*ethanol leaf extract exhibited antibacterial activity against foodborne pathogen (e.g., *Listeria monocytogenes, P. aeruginosa)* with significant zone of inhibition (Widjajakusuma et al., 2019). Ethyl acetate extract of *S. stocksii* leaves exhibited antibacterial activity against six bacterial strains (e.g. *P. aeruginosa*), determined by disk diffusion method (Eganathan et al., 2012). *S. samarangense* leaves essential oil extract exhibited strong antibacterial activity against both gram‐positive (e.g. *B*. *cereus*) and gram‐negative bacteria (e.g. *E*. *coli*) with significant zone of inhibition, determined by agar well diffusion method (Reddy et al., 2011). Methanol and aqueous extracts of *S. zeylanicum* bark and leaves exhibited antibacterial activity, and this activity was independent of gram reaction (Shilpa et al., 2014). Its fruits extract also exhibited antibacterial activity (Shilpa & Krishnakumar, 2015).

### 5.4 Antifungal activity

*S. aromaticum* oil (clove oil) showed strong antifungal activity against *Trichophyton mentagrophytes*, *Trichophyton rubrum*, *Microsporum gypseum,* and *Microsporum canis,* and Eugenol was the most effective antifungal constituent of clove oil (Park et al., 2007). *S*. *australe* (leaf), *S. jambos* (leaf), *S. luehmannii* (leaf and fruit) methanol extracts showed potent activity against fungal growth through inhibiting the growth of human dermatophytes (Noé et al., 2019). Ethyl acetate extract of *S. benthamianum* leaves showed antifungal activity by inhibiting the growth of *Proteus vulgaris* at the MIC of 100 μg/ml (Kiruthiga et al., 2011). Ethyl acetate extract of *S. calophyllifolium* leaves showed maximum zone of inhibition against *T*. *mentagrophytes* and expressed its antifungal activity (Vignesh et al., 2013). Methyl acetate extract of *S. caryophyllatu* leaves exhibited antifungal activity against three fungal strains (e.g. *Alternaria alternata*), assessed in disk diffusion method (Annadurai et al., 2012). Dichloromethane, ethanol, and water extracts of *S. cordatum* leaves exhibited the best antifungal activity with MIC values of 0.20, 0.39, and 0.78 mg/ml, respectively (Mulaudzi et al., 2012). The aqueous and dichloromethane–methanol extracts of bark exhibited antifungal activity producing inhibition effect against several bacterial pathogens, determined by using the microtiter plate dilution assay (Nciki et al., 2016). Ethyl acetate extract of *S. densiflorum* leaves exhibited antifungal activity against three fungal species (*Aspergillus niger*), determined by disk diffusion method (Eganathan et al., 2012). *S. malaccense* methanol leaf extract exhibited selective antifungal activity against *Microsporum canis*, *Trichophyton rubrum* and *Epidermophyton floccosum* through inhibiting their growth (Locher et al., 1995). Ethyl acetate extract of *S. stocksii* leaves exhibit antifungal activity against three fungal species (*A*. *niger*), determined by disk diffusion method (Eganathan et al., 2012; Table 3).

### 5.5 Anticancer activity

Anticancer substances are those substances which exhibited its cytotoxic effect against different cancer cell lines (Table 3). In vitro anticancer activity of leaf hexane and methanol extracts and its isolated two compounds (eucalyptin and epibetulinic acid) of *S. alternifolium* was showed significant activity (IC50 values 8.177 and 2.687 µg/ml) when compared with others human cancer cell lines (MCF‐7) and human prostate cancer cell lines (DU‐145). *S. aromaticum* bud essential oil extract was evaluated to determine the cytotoxicity using MTT assay, colony formation assay, and Annexin V‐FITC assay against the thyroid cancer cell line (HTh‐7) and found that the extract showed significant anticancer activity (Nirmala et al., 2019). Methanol and aqueous extracts of *S. austral* fruits were potent inhibitors of cell proliferation against CaCo2 and HeLa cancer cells, determined by an MTS‐based cell proliferation assay (Jamieson et al., 2014). Ethyl acetate extract of *S. benthamianum* leaves showed higher activity on Hep2 cells by inhibiting the cell growth, determined by MTT assay (Kiruthiga et al., 2011). n‐Hexane methanol extract of *S. campanulatum* leaves showed antiproliferative activity on human colon cancer (HCT 116) cell line (Memon et al., 2015). Ethyl acetate extract of *S. calophyllifolium* leaves showed anticancer activity; the extract has higher cytotoxic activity against Hep2 cell lines (Vignesh et al., 2013). Its methanol extract of bark showed antiproliferative and cell death‐inducing ability analyzed by using MCF‐7 breast cancer cell (Chandran et al., 2018). Ethyl acetate extract of *S. caryophyllatum* leaves exhibited maximum cell inhibition at higher concentration on cell viability of Hep2 cell lines determined by MTT assay (Annadurai et al., 2012). Ursolic acid and (+)‐2,3‐dihydrosideroxylin isolated from the leaves of *S. corticosum* were evaluated for their cytotoxicity against the HT‐29 human colon cancer cell line, and it was reported that both the compounds produced cytotoxic effect against the cancer cell line (Ren et al., 2018). *S. cumini* ethanol fruit extract showed anticancer property through exhibiting a significant dose‐dependent inhibitory effect on cancer cell lines or on AML (acute myeloid leukemia, immature monocytes) cell line (Afify et al., 2011). *S. fruticosum* methanol seed extract showed anticancer property through exhibiting a significant dose‐dependent inhibitory effect on Ehrlich's Ascite cell (EAC)‐induced Swiss albino mice (S. Islam et al., 2013). *S. gratum* leaf extract produced cytotoxicological effects on gastric and breast cancer cell lines (e.g., Kato‐III, NUGC‐4, MCF‐7, MDA‐MB‐231), determined by MTT assay (Rocchetti et al., 2019; Stewart et al., 2013). *S. guineense* methanol plant extract showed anticancer activity against triple‐negative breast cancer and colon cancer cells through inhibiting Wnt‐signaling and proliferation of Wnt‐dependent tumors (Koval et al., 2018). *S. jambos* exhibited anticancer activity (Chua et al., 2019). Ethanol and chloroform bark extracts of *S. johnsonii* showed cytotoxicity against several cancer cell lines such as HepG2 and MDA‐MB‐231(Harris et al., 2011; Setzer et al., 2001). *S. lineatum* leaf extract exhibited antiproliferative effects on HUVEC (human umbilical vein endothelial cells) and cytotoxic effect on Hela (cervical cancer cell line), determined by MTT assay (Castillo et al., 2017). Methanol and aqueous extracts of *S. luehmannii* fruits were potent inhibitors of cell proliferation against CaCo2 and HeLa cancer cells, determined by an MTS‐based cell proliferation assay (Jamieson et al., 2014). Methanol extract of *S. malaccense* fruit exhibited anticancer activity against MCF‐7 and MDA‐MB‐231 (Itam & Anna, 2020). *S. mundagam* methanol bark extract exhibited anticancer activity inducing toxicity in MCF7 breast cancer cells (Chandran et al., 2020). *S. polyanthum* hydro‐methanol leaf extract exhibited anticancer activity against 4T1 and MCF‐7 mammary carcinoma cells (Nordin et al., 2019). *S. paniculatum* fruit extract exhibited anticancer activity by reducing cell viability in both MiaPaCa‐2 and ASPC‐1 pancreatic cancer cells (Vuong et al., 2014). Its leaf extract also exhibited cytotoxic effects against MCF‐7 breast adenocarcinoma and MDA‐MB‐231 breast cancer cell lines (Rocchetti et al., 2019). Three compounds (2`,4`‐dihydroxy‐3`,5`‐dimethyl‐6`‐methoxychalcone; 2`,4`‐dihydroxy‐3`‐methyl‐6`‐methoxychalcone (stercurensin); and 2`,4`‐dihydroxy‐6`‐methoxychalcone (cardamonin)) isolated from the methanol extracts of the pulp and seeds of the fruits of *S*. *samarangense* exhibited cytotoxic effect against human colon cancer cell line (SW‐480; Simirgiotis et al., 2008).

### 5.6 Antidiabetic activity

The substances which are used to treat diabetes mellitus through altering the blood glucose level in blood are called antidiabetic or hypoglycemic or antihyperglycemic agents. Many compounds of several classes isolated from different species of the genus exhibited antidiabetic activity. For instance, flavanone 5‐O‐methyl‐4′‐desmethoxymatteucinol 2 exhibited antihyperglycemic effects through altering the blood glucose level (Resurreccion‐Magno et al., 2005). Aqueous extract of *S. alternifolium* seeds at a dose of 50 mg/kg exhibited antihyperglycemic activity and produced maximum fall of 83% in the blood glucose level in diabetic rat (Kasetti et al., 2010). Bioactive compounds (e.g., 4‐hydroxybenzaldehyde, myricetin‐3‐O‐rhamnoside) of leaf extract of *S. aqueum* effectively increased adipogenesis, stimulated glucose uptake, and also increased adiponectin secretion and showed antidiabetic potentiality (T. Manaharan et al., 2013). *S. aromaticum* essential oil from bud extract exhibited a stronger antidiabetic activity with 95.30% inhibition of α‐amylase (Tahir et al., 2016). Methanol extract of *S. calophyllifolium* barks reduced the blood glucose level and exhibited antidiabetic effect in streptozotocin‐nicotinamide (STZ‐NA)‐induced diabetic rats (Chandran et al., 2016). Hexane fraction of *S. caryophyllatum* fruits exhibited significantly high antiamylase activity with IC_50_ value of 2.27 ± 1.81 μg/ml and also exhibited antidiabetic effects (Wathsara et al., 2020). Aqueous extract of *S. cordatum* leaves exhibited strong antidiabetic effects on streptozotocin (STZ)‐induced diabetic rats through lowering the blood glucose levels (Deliwe & Amabeoku, 2013). Ethyl acetate and methanol extracts of *S. cumini* seed exhibited the antidiabetic activity against STZ‐induced diabetic rats. Both extracts produced significant (*p* < .05) reduction in blood glucose level (A. Kumar et al., 2013). Its ethanol leaf extract exhibited the antidiabetic activity against alloxan‐induced diabetic rats by reducing blood glucose level (Schoenfelder et al., 2010). *S. densifloru m*ethanol leaf extract exhibited significant reduction in the elevated blood glucose level where the percentage of activity at a concentration of 200 mg/kg b.w was higher than the standard drug metformin (MK et al., 2013). Ethanol fruit extract also exhibited antidiabetic activity in STZ‐ and nicotinamide (NA)‐induced diabetic rats (Krishnasamy et al., 2016). *S. grande* bark extract showed antidiabetic properties (Myint, 2017). *S. guineense* aqueous leaf extract exhibited in vitro antidiabetic activity by effecting on glutathione levels within HepG2 cells and inhibiting P‐glycoprotein efflux (Ezuruike et al., 2019). *S. jambos* aqueous leaf extract exhibited antidiabetic activity by reducing blood glucose level in diabetes genetic mouse models (db/db; Gavillán‐Suárez et al., 2015). Aqueous bark extract also exhibited antidiabetic activity by reducing blood glucose level at a high dose determined by using normoglycemic (in fasted and nonfasted states) and STZ‐induced diabetic rats (Hettiarachchi et al., 2004). *S. luzonense* ethanol bark extract exhibited antihyperglycemic activity on alloxan‐induced rats by reducing the blood sugar with an optimal dose of 300 mg/kg b.w (Walean et al., 2020). *S. mundagam* methanol bark extract exhibited antidiabetic activity by reducing blood glucose level in STZ‐NA‐induced diabetic rats (Chandran et al., 2017). *S. paniculatum* methanol and aqueous fruit extract exhibited antidiabetic activity by reducing blood glucose level in STZ‐induced diabetic rats (Konda et al., 2019). *S. polyanthum* aqueous leaf extract exhibited antidiabetic activity through reducing blood glucose level in alloxan‐induced diabetic rats (Widjajakusuma et al., 2019). Its methanol leaf extract also exhibited antihyperglycemic effects on STZ‐induced diabetic rats (Widyawati et al., 2015). Vescalagin, a compound isolated from *S. samarangense* fruit, exhibited antidiabetic activity against high‐fructose diet (HFD)‐induced diabetic Wistar rats by lowering the plasma insulin and C‐peptide levels (Shen & Chang, 2013). In another study, a compound isolated from leaves of *S. samarangense* exhibited antihyperglycemic effects on alloxan‐induced diabetic rats (Resurreccion‐Magno et al., 2005).

### 5.7 Antidiarrheal activity

Antidiarrheal agents are fiber‐forming substances which are used to treat or relieve the symptoms of diarrhea (S. Ahmad et al., 2020; Ansari et al., 2017). *S. cordatum* leaf aqueous extract reduced the number of diarrheal episodes, decreased the stool mass, and delayed the onset of castor oil‐induced diarrhea in mice (Deliwe & Amabeoku, 2013). Its methanol extract of fruit pulp and seed extract exhibited the antidiarrheal activity by reducing the number of wet stools, total stools, and onset time in castor oil‐induced rats (Sidney et al., 2015b). *S. guineense* ethanol leaf extract exhibited antidiarrheal activity in mice significantly (*p* < .05) by inhibiting the intrinsic small intestinal propulsion and itopride‐induced propulsive activity (Ezenyi & Igoli, 2018). Isolated compounds from *S. myrtifolium* ethanol leaf extract exhibited antidiarrheal and antispasmodic potentiality for selected therapeutic effect (Memon et al., 2020). Hexane extract of *S. samarangense* leaves exhibited spasmolytic activity by relaxing the high K+‐induced contractions and also decreased the Ca++ dose–response in a dose‐dependent manner (Ghayur et al., 2006; Table 3).

### 5.8 Hepatoprotective activity

The ability of a substance to prevent damage or injury of liver is called antihepatotoxicity or hepatoprotective activity. For example, CCl4 causes hepatotoxic effect through various appliances, and the antihepatotoxic substances such as flavonoids (e.g. myricetin) as natural resource of plants counteract that effects by reducing the injury level by different facts (Sobeh et al., 2018). *S. aqueum* leaf extract showed hepatoprotective activity by reducing the elevated levels of ALT, AST, total bilirubin (TB), total cholesterol (TC), and triglycerides (TG) in rats with acute CCl_4_‐induced intoxication. In addition to reducing the high MDA level, the extract noticeably restored GSH and SOD to the normal control levels in liver tissue homogenate and counteracted the deleterious histopathologic changes in liver after CCl_4_ injection (Sobeh, Mahmoud, et al., 2018, 2018). *S. aromaticum* clove oil extract showed in vitro hepatoprotective potential against CCl_4_‐induced hepatotoxicity using rat liver slice culture (LSC) model (Hina et al., 2017). *S. cumini* seed powder with HCHF (high carbohydrate high fat) food supplementation reduced the high‐fat diet‐induced fatty liver or hepatic steatosis in rats. It is also noted that its seed powder prevented the rise of plasma TC and TG levels (Ulla et al., 2017). Methanol extract of *S. densifloru* fruits showed antihyperlipidemic activity (Krishnasamy et al., 2016). *S. jambos* ethanol leaf extract exhibited hepatoprotective activity in a rat model of CCl_4_‐induced liver damage (Islam et al., 2012). *S. samarangense* methanol leaf extract showed hepatoprotective activity by reducing liver injury using CCl_4_‐inducedrats (Sobeh et al., 2018).

### 5.9 Others

The addition of polyphenolic‐rich extracts from the leaves of *S. anisatum* to the culture media exerted supreme cytotoxic effect through reducing cell viability of the following cancer cell lines: HT‐29, AGS, BL13, and HepG2, in a dose‐dependent manner (Sakulnarmrat et al., 2013). *S. aromaticum* leaves contain eugenol that inhibited biofilm formation and reduced preformed biofilm of *P. gingivalis* at different concentrations (Zhang et al., 2017). Its bud methanol extract and hydrodistillate showed larvicidal and ovicidal potentiality against third‐instar larvae and eggs of *B. procera* (Hong et al., 2018). Its essential oils displayed antiulcer activities in the rat models of indomethacin and ethanol‐induced ulcer (Santin et al., 2011). Also bud extracts exhibited antituberculosis activity, the proportion of inhibition for *M. tuberculosis* H37Rv, was found to be dose dependent (Kaur & Kaur, 2015). Its ethanol bud extract also showed anthelmintic activity (Patil et al., 2014). n‐Hexane extract of *S. campanulatum* leaves suppressed expression of VEGF in endothelial cells. It inhibited angiogenesis and tumor growth in nude mice and showed antiangiogenesis effect and antitumor activity (Aisha et al., 2013). *S. cordatum* bark extracts significantly lowered the effect of the mutagen mitomycin C (MMC) and showed antimutagenic effects (Verschaeve et al., 2004). Its leaf extracts exhibited significant leishmanicidal activity with acceptable SI values (SI ≥10) to determine their potential lethality or safe therapeutic application against rat skeletal myoblast L6 cell (Bapela et al., 2017). *S. cordatum* also showed antiplasmodial activity through inhibiting the growth of the chloroquine‐resistant Dd2 malaria parasite strains (Nondo et al., 2015). *S. cumini* ethanol leaf extracts exhibited antinociceptive effect through showing marked inhibition (*p* < .01 or *p* < .001) of glutamate‐induced orofacial nociception (38.8, 51.7, and 54.7%) when compared with the control group (Quintans et al., 2014). Its aqueous leaf extract showed antiallergic properties inhibiting the paw edema induced by C48/80, a potent mast cell degranulator, to an extent comparable to the effect of promethazine, a classical antihistaminic used to relieve symptoms of allergic reactions (Brito et al., 2007). Its methanol and aqueous bark extract exhibited anthelmintic effect using *Pheretima posthuma* as the animal models, and the effect of the extract is comparable to that of standard drug, Albendazole (Kavitha et al., 2011). Its ethanol extract of seeds at the dose of 250mg/kg and 500 mg/kg inhibited the Freund's complete adjuvant (FCA) induced arthritis in rats (E. Kumar et al., 2008). Carbon tetrachloride soluble fraction of *S. fruticosum* leaf extract showed significant lethality having the LC_50_ value 0.65µg/ml. It also exhibited thrombolytic activity (Chadni et al., 2014). Ethanol extract of *S. formosum* leaves showed antiallergic activity through inhibiting the allergic symptoms to a significant extent in a dose‐dependent manner, examined with a mouse model of chicken ovalbumin (cOVA)‐induced food allergy (Nguyen et al., 2018). *S. guineense* leaf extract exhibited antimalarial activity in mice through the suppression of parasite (e.g., malaria parasite) at doses of 600 and 400 mg/kg (Tadesse & Wubneh, 2017). Its hydro‐alcoholic leaf extract exhibited in vivo antihypertensive activity in a rat model by reducing blood pressure and also showed in vitro vasodepressor activity by relaxation of aorta precontracted with KCl (Ayele et al., 2010). Its chloroform stem bark extract exhibited antituberculosis activity assessed by using the Mycobacterium Growth Indicator Tube (MGIT) method (Oladosu et al., 2017). Its ethanol fruit extract exhibited cytotoxicity and antihelmintic activity against *Artemia salina* and *Pherithema posthuma* pathogen, respectively (Maregesi et al., 2016). Methanol stem bark extract inhibited intrinsic contractions of rabbit and also produced sustained hypotension in anaesthetized rats and reduce systolic, diastolic blood pressure exhibiting antispasmodic activity (Malele et al., 1997). *S. jambos* hydro‐alcoholic leaf extract exhibited significant antinociceptive and analgesic activity in a rat model (Avila‐Peña et al., 2007). Its hydro‐ethanol leaf extract exhibited antiulcerogenic activity in a rat model reducing gastric injury induced by HCl/ethanol (Donatini et al., 2009). Its chloroform‐methanol fruit extract exhibited antitumor activity in Ehrlich tumor‐bearing mice reducing the tumor growth (Tamiello et al., 2018). Its fruit extract exhibited cytotoxic effects on melanoma cells by 3‐(4,5‐dimethylthiazol‐2‐yl)‐2,5‐diphenyltetrazolium bromide assay (Li et al., 2015). Methanol bark extract exhibited antileukemic activity with the survived cell percentage (HL‐60 cells) of 12.7% among tested sample (Pardede et al., 2020). *S. lanceolatum* leaf essential oil exhibited larvicidal effect against anopheline and culicine species (Benelli et al., 2018). *S. legatii* acetone leaf extract showed antibiofilm activity through reducing biofilm formation (I. M. Famuyide et al., 2019). Its acetone leaf extract also showed anti‐quorum sensing activity determined by inhibition of quorum sensing (QS)‐controlled violacein pigment production in *Chromobacterium violaceum* (I. Famuyide et al., 2019). *S. malaccense* aqueous bark extract exhibited antiviral activity against Herpes Simplex Virus‐1 and 2 and inhibited the growth of these viruses (Locher et al., 1995). Its leaven‐hexane, ethyl acetate, and methanol extract exhibited cytotoxicity properties on brine shrimp (Itam & Anna, 2020). Methanol and water extracts of *S. myrtifolium* leaves exhibited antidermatophytic activity against *Trichophyton rubrum* and *T. interdigitale* dermatophytes. These extraction also exhibited fungicidal activity and cytotoxic activity against several isolates of *T. tonsurans* determined by spread plate method and epithelial (Vero) cell line of monkey, respectively (Sit et al., 2018). *S. samarangense* methanol leaf extract exhibited analgesic and CNS depressant activities, determined by writhing test and reduction of locomotor and exploratory activities in the open field and hole cross tests, respectively (Mollika et al., 2014). Its fruit extracts exhibited antiapoptotic properties against STZ‐induced pancreatic ß‐cell damage in diabetic rats. Ethanol bark extracts exhibited significant anthelmintic activity against five worms (Gayen et al., 2016). Its fruit extracts exhibited anti‐acne activity (Goni et al., 2021; Sekar et al., 2017). Leaf extracts of n‐hexane and ethyl acetate fraction exhibited anti‐obesity effect on Wistar rat through inhibiting body weight. *S. zeylanicum* leaf essential oil exhibited potent larvicidal effect against larvae of mosquitos (e.g. *Aedes albopictus, Anopheles subpictus*; Govindarajan & Benelli, 2016).

## 6 CONCLUSION

In this review article, we have tried to present the organized information on traditional uses, phytochemical constituents, and mechanism‐based pharmacological activities of plants belonging to the genus *Syzygium*. The collected data from various literatures turned evident that the plants of the genus *Syzygium* had prominent traditional uses along with potential pharmacological properties. The reviewed investigation revealed many different pharmacological activities obtained from several organic extracts, essential oils, and compounds isolated from *Syzygium* species. Some of the activities such as antioxidant, anti‐inflammatory, antibacterial, anticancer, hepatoprotective, antidiarrheal activities, etc. were exhibited as pharmacological activities. Many of them also applied on the animal model for better result along with in vitro screening. The in vivo data complied with the data obtained from the in vitro studies. However, only few species of the genus were studied for their phytochemical constituents that could arbitrate pharmacological activities and still many are unidentified and multiple gaps in the knowledge exist for the lack of isolated compounds. Many of the implemented pharmacological studies were limited to the in vitro screening, many of the pharmacological studies were not correlated with traditional uses, and also many of the animal model‐based investigations were done without mentioning their detailed mechanisms of action. This review article linked the phytochemical constituents to pharmacological activities and provided perception of the biological potential of the genus *Syzygium*. Study of pharmacological activities delivered supportive evidence for therapeutic effect of this genus. However, many members of the genus *Syzygium* need more inclusive studies regarding phytochemical constituents and mechanism‐based pharmacological activities. Also, in vitro and in vivo animal studies are necessary to ascertain the safety, clarification of the effective doses, and the mechanisms of action before future clinical studies.

## CONFLICT OF INTEREST

All the authors have read and approved the manuscript for this journal. They do not have any conflict of interest.

## AUTHOR CONTRIBUTIONS

**A. B. M Neshar Uddin:** investigated the study and provided resources. **Farhad Hossain:** performed data curation, investigated the study, contributed to software, and wrote the original draft. **A. S. M. Ali Reza:** conceived and designed the review. **Mst Samima Nasrin:** interpreted the data and drafted the manuscript. **A. H. M. Khurshid Alam:** corrected the manuscript and interpreted the data.
